# Visualization and Quantification of Genetically Adapted Microbial Cells During Preculture

**DOI:** 10.3389/fmicb.2021.693464

**Published:** 2021-07-14

**Authors:** Hyun Ju Kim, Haeyoung Jeong, Sang Jun Lee

**Affiliations:** ^1^Department of Systems Biotechnology, Institute of Microbiomics, Chung-Ang University, Anseong, South Korea; ^2^Infectious Disease Research Center, Korea Research Institute of Bioscience and Biotechnology (KRIBB), Daejeon, South Korea

**Keywords:** adaptation, real-time, visualization, lag phase, *Escherichia coli*, succinate, BL21(DE3), *kgtP*

## Abstract

As culture history is known to affect the length of the lag phase and microbial cell growth, precultures are often grown in the same medium as the main culture for physiological adaptation and to reduce a prolonged lag time in some microbial cells. To understand the adaptation process of microbial cells during transfer from Luria–Bertani medium to minimal medium, we used the growth of *Escherichia coli* BL21(DE3) in succinate minimal medium as a model system. We observed that only one or two sequential transfers from minimal medium to fresh minimal medium accelerated the growth rate of BL21(DE3) cells. In addition, the number of large colonies (diameter ≥0.1 cm) on succinate agar increased with the number of transfers. Genome and transcript analyses showed that the C-to-T point mutation in large colony cells converted the inactive promoter of *kgtP* (known to encode α-ketoglutarate permease) to the active form, allowing efficient uptake of exogenous succinate. Moreover, we visualized the occurrence of genetically adapted cells with better fitness in real time and quantified the number of those cells in the microbial population during transfer to the same medium. Fluorescence microscopy showed the occurrence and increase of adapted mutant cells, which contain intracellular KgtP-fused green fluorescent proteins, as a result of the C-to-T mutation in the promoter of a fused *kgtP–sfgfp* during transfer to fresh medium. Flow cytometry revealed that the proportion of mutant cells increased from 1.75% (first transfer) to 12.16% (second transfer) and finally 70.79% (third transfer), explaining the shortened lag time and accelerated growth rate of BL21(DE3) cells during adaptation to the minimal medium. This study provides new insights into the genetic heterogeneity of microbial populations that aids microbial adaptability in new environments.

## Introduction

Upon exposure to new environmental conditions, microbial cells need some time to optimize gene expression to synthesize building blocks for cell division; this is known as the lag phase (Monod, 1949; Buchanan and Solberg, 1972; Pirt, 1975; Srivastava and Volesky, 1990). Cellular growth in the lag phase is delayed; cells in this phase take longer to reach the normal or exponential rate of cell growth, presumably because it takes time to fully achieve the expression of metabolic genes for new substrates (Dean, 1957) or to take up trace metals from the extracellular milieu (Rolfe et al., 2012). Despite the fact that it is difficult to obtain sufficient samples from poor microbial growth in the lag phase, the dynamics of promoter activity in the lag phase of *Escherichia coli* have been studied during metabolic adaptation (Madar et al., 2013). The lag phase has been known to be extensively relevant to the study of bacterial evolution (Adkar et al., 2017), antibiotic tolerance (Madar et al., 2013; Fridman et al., 2014), and host–microbe interactions (Bertrand, 2014).

Previous studies showed that the bacterial lag phase is not simply a gradual change in the entire population, which could be due to non-genetic phenotypic heterogeneity (van Boxtel et al., 2017). For example, a subpopulation of *Lactococcus lactis* was shown to be fit to partake in a second growth phase during diauxic shift (Solopova et al., 2014). Similarly, glycerol-dependent metabolic persistence in *Pseudomonas putida* cells was suggested to occur via the stochastic repression of glycerol-metabolizing genes (Nikel et al., 2015). Further, the composition of the culture medium influences both gene expression in the population and the emergence of phenotypic variants (Smith et al., 2018).

Fluorescently labeled cells can be mixed to observe microbial population dynamics (Reyes et al., 2012). By analyzing fluorescence in a single cell, the physiological phenomena of microorganisms can be visualized. *E. coli* was grown in a microfluidic device to monitor the expression of the fluorescent protein, showing that the microorganism can produce the protein even in the lag phase (Gefen et al., 2014). Viable but non-culturable and persister cells could be distinguished through a fluorescence-based live/dead staining after antibiotic treatment in microfluidics (Bamford et al., 2017).

The lag time and growth rate of cells can be measured simultaneously by scanning the growth of a large number of colonies, reporting that there were subgroups with prolonged lag time after antibiotic stress (Levin-Reisman et al., 2010, 2014). Diversity of lag time in microbial populations provides resistance to stressful factors such as antibiotics (Fridman et al., 2014; Moreno-Gamez et al., 2020). The evolution of antibiotic-resistant lineages was visualized on microbial evolution and growth arena plates, and the associated genetic mutations were characterized (Baym et al., 2016).

In our study, molecular evolution was fluorescently visualized in real time during microbial adaptation to new nutritional environments. *E. coli* BL21(DE3) was harnessed as a model system to understand how bacterial populations adapt to grow in new nutritional environments. In addition to the fact that the length of the lag phase of cells depends on culture media, culture history, and seed size, we showed the occurrence of genetically adapted cells in a microbial population, which can contribute to the shortened lag phase during transfer to the same culture medium.

## Materials and Methods

### Bacterial Strains

The bacterial strains, phages, and plasmids used in this study are shown in Table 1. The *E. coli* K-12 BW25113 strain was obtained from the Coli Genetic Stock Center (CGSC) at Yale University (New Haven, CT, United States). The *E. coli* B strain BL21(DE3) was purchased from Invitrogen (Carlsbad, CA, United States). The Keio collection (Baba et al., 2006), individual gene knockout mutants of *E. coli* K-12 BW25113, was purchased from Open Biosystems (Lafayette, CO, United States). The mutations were transferred to other strains via standard P1 transduction (Miller, 1992). Whenever needed, the plasmid pKD46 was used to transform the *E. coli* strains with polymerase chain reaction (PCR) products via homologous recombination.

**TABLE 1:** Bacterial strains, phage, and plasmids used in this study.

Name	Relevant genotypes and characteristics	References or sources
Strains		
BW25113	*E. coli* K-12, Δ*(araD-araB)567* Δ*lacZ4787(::rrnB-3)* λ^–^ *rph*^–1^ Δ*(rhaD-rhaB)568 hsdR514*	CGSC
BL21(DE3)	F^–^ *ompT gal dcm lon hsdSB*(r_B_^–^ m_B_^–^) λ(DE3 [*lacI lacUV5*-T7 gene 1 *ind1 sam7 nin5*])	Invitrogen
JW5409	BW25113, Δ*yfiP*:: KmR	Keio collection
HK565*	BL21(DE3), *kgtP*^(–8C→T)^	This study
HK567*	BL21(DE3), Δ*kgtP*::KmR	This study
HK569*	BL21(DE3), Δ*kgtP*::KmR, *dcuS*^rev^	This study
HK651	BL21(DE3), *kgtP*^WT^ Δ*yfiP*::KmR	This study
HK652	BL21(DE3), *kgtP*^(–8C→T)^ Δ*yfiP*::KmR	This study
HK654	HK565, *kgtP*^WT^ Δ*yfiP*::KmR	This study
HK655	HK565, *kgtP*^(–8C→T)^ Δ*yfiP*::KmR	This study
HK1020	BL21(DE3), *kgtP*^WT^-GGGGS linker-sfGFP	This study
HK1022	HK565, *kgtP*^(–8C→T)^-GGGGS linker-sfGFP	This study
Phage		
P1 *vir*	*vir* mutations	S. Adhya
Plasmids		
pCP20	Temperature-sensitive plasmid with an FLP recombinase capable of recognizing the FRT sequence, ApR	CGSC
pKD46	Temperature-sensitive plasmid with lambda recombinase inducible by L-arabinose, ApR	CGSC
pJET1.2	Cloning plasmid, ApR	Thermo Fisher Scientific

*Whole-genome sequencing was performed to identify a point mutation (− 8C→T) in the *kgtP* gene.

### Culture Conditions

Luria–Bertani (LB) broth and 5 × M9 salts were purchased from Becton Dickinson (Sparks, MD, United States). Glucose, sodium succinate dibasic, and α-ketoglutaric acid disodium salt dehydrate were purchased from Sigma–Aldrich (St. Louis, MO, United States). The M9 minimal medium is composed of the following: 0.8 g/L NH_4_Cl, 0.5 g/L NaCl, 7.5 g/L Na_2_HPO_4_⋅2H_2_O, 3 g/L KH_4_PO_4_, 0.2 g/L MgSO_4_⋅7H_2_O, 0.1 g/L CaCl_2_, and 3 g/L carbon source (e.g., glucose, succinate, and α-ketoglutarate). A single colony of *E. coli* was grown in 5 mL LB broth at 37°C with shaking at 180 revolutions per minute (rpm) for 12 h. Bacterial cultures were harvested by centrifugation and washed twice with phosphate-buffered saline (PBS). Then, the cells were resuspended in 5 mL of PBS, and 500 μL of the cell suspension, with approximately 10^8^ cells, was inoculated into 50 mL of M9 succinate (0.3%) minimal media. Cell cultures were incubated at 37°C with shaking at 180 rpm. Cell growth was measured every 3 h according to the optical density (OD) at 600 nm using an Ultraspec 8000 spectrophotometer (GE Healthcare, Uppsala, Sweden). The period until OD at 600 nm reached 0.05 was calculated as the lag time. The specific growth rate (μ; h^–1^) for the exponential phase was calculated by the change of logarithm value of OD_600__nm_ per time (h), which is μ = (LnOD_2_ - LnOD_1_)/(*t*_2_ - *t*_1_) (Widdel, 2007).

### Genome Analysis

The *E. coli* HK565 strain was grown aerobically in LB medium at 37°C for 12 h. Genomic DNA was isolated using the Wizard Genomic DNA Purification Kit (Promega, Madison, WI, United States). Library construction using Illumina TruSeq DNA Sample Preparation Kit v2 (Illumina, San Diego, CA, United States) and 101 Cycle Paired-End Sequencing using the Illumina HiSeq 2000 system were performed according to the manufacturer’s protocol at the National Instrumentation Center for Environment Management (NICEM, Seoul, South Korea).

Read preprocessing (quality trim limit 0.01, one ambiguous nucleotide allowed per read, and minimum read length 50 np), reference mapping, and fixed ploidy variant detection (including indels and structural variants detection) were conducted using CLC genomics Workbench 7.5.1 (QIAGEN, Hilden, Germany). The complete genome sequence of *E. coli* BL21(DE3) (NC_012971.2) was used as a reference. Genome sequencing data were deposited in the NCBI BioProject database under the accession number PRJNA529313 (SRA SRX5608579, SRX5608580, and SRX5608581).

### Transcript Analysis

Bacterial cultures were treated with RNAprotect Bacteria Reagent (QIAGEN) to quench RNA degradation, and total RNA was isolated using an RNeasy Mini Kit (QIAGEN) according to the manufacturer’s instructions. The transcription start site was analyzed using the 5′ terminal rapid amplification of cDNA ends (5′-RACE) using a 5′-Full RACE core kit (TaKaRa Bio, Inc., Kyoto, Japan) according to the manufacturer’s instructions. cDNA was synthesized from the total RNA using a 5′-phosphorylated DNA primer (RT_5P_2) (Supplementary Table 1). Single-stranded cDNA was treated with RNase H for RNA degradation. Subsequently, single-stranded cDNA was circularized using T4 RNA ligase at 15°C for 18 h and amplified by PCR with S1 and A1 primers (Supplementary Table 1). PCR products were cloned into the pJET1.2 cloning vector (Thermo Fisher Scientific, Waltham, MA, United States). The transcription start point was confirmed via DNA sequencing of the cloned sequences. The expression levels of the *kgtP* gene were measured using quantitative reverse transcriptase (RT)–PCR: primers were designed at the Universal Probe Library Assay Design Center,^1^ and reactions were carried out on a LightCycler 96 (Roche Diagnostics, Mannheim, Germany) using the RealHelix^TM^ quantitative RT-PCR kit (NanoHelix, Daejeon, South Korea). RT-PCR reactions were obtained as per the following steps under the following conditions: cDNA synthesis (50°C, 40 min), denaturation (95°C, 12 min), and amplification for 40 cycles (95°C, 20 s; 60°C, 1 min). The raw fluorescence data were normalized against the 16S ribosomal RNA expression level. All primers used are shown in Supplementary Table 1.

### Genomic Construction

For the genetic complementation experiments, Δ*yfiP*::KmR cassettes obtained from the Keio collection were electroporated into either *E. coli* BL21(DE3) or HK565 strains, both harboring pKD46 plasmids after lambda recombinase was fully induced by L-arabinose, to tag *kgtP*^(–8C →T)^ and *kgtP*^WT^ mutations. Subsequently, the P1 lysate of the resulting cells was used to transduce kanamycin marker-tagged *kgtP*^(–8C →T)^ and *kgtP*^WT^ mutations into the BL21(DE3) or HK565 strains to generate the HK651, HK652, HK654, and HK655 strains.

To introduce the translational fusion *sfgfp* gene (Pedelacq et al., 2006) downstream of the *kgtP* gene, we inserted the *sfgfp*-CmR cassettes downstream of the *kgtP* gene with linker sequences (GGGGS). The *kgtP* gene partial fragments, *sfgfp*-CmR, and the *pssA* gene partial fragments were amplified using PCR; the three were fused using overlap PCR with six primers (Supplementary Table 1) and were electroporated into *E. coli* BL21(DE3) or HK565 cells harboring plasmid pKD46 after lambda recombinase was fully induced by L-arabinose, generating the HK1020 and HK1022 strains, respectively.

### Fluorescence Measurement

The expression of cellular fluorescent proteins in the cells was observed via fluorescence microscopy (L5 filter; excitation 480/40 nm; emission 527/30 nm) using a CCD camera (DM2500 LED; Leica, Wetzlar, Germany).

To analyze the fluorescence intensity of the bacterial cells at a single-cell level, the flow cytometer CyFlow Cube 6 system (Sysmex-Europe GmbH, Norderstedt, Germany) was used, which is equipped with a 488-nm blue solid-state laser. Fluorescence intensity was detected via the channel FL1 at 530 ± 40 nm for sfGFP. Samples were diluted 100-fold to obtain 10^5^ counts (5,000 particles/s), and the flow was set to 0.5 μL/s during measurement.

Total fluorescence amounts of the sfGFP proteins expressed in the HK1020 and HK1022 strains were measured respectively, using a TECAN Infinite 200 PRO plate reader (excitation 485/20 nm; emission 535/25 nm; gain = 40, top reading) and TECAN i-control software (TECAN, Salzburg, Austria).

## Results

### Factors Affecting the Duration of the Lag Phase in the Growth of *E. coli* Strains

**FIGURE 1:**
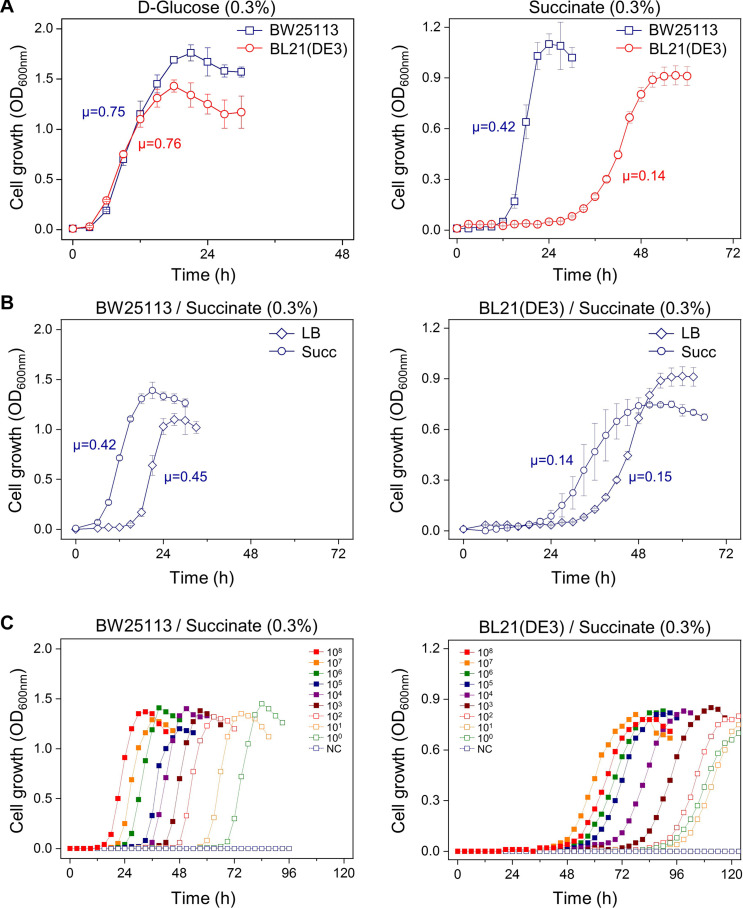
Factors affecting the length of the lag phase of *E. coli* BW25113 and BL21(DE3). **(A)** Different carbon sources; cells grown in LB broth were washed and transferred to M9 minimal medium containing D-glucose or succinate. **(B)** Different preculture medium; cells grown in LB and M9 succinate medium were washed and transferred to M9 succinate medium. **(C)** Different inoculum size; cells grown in LB broth were washed and transferred to M9 succinate medium. NC, no cells were inoculated. The unit of specific growth rate (μ) is h^–1^.

We tested whether the carbon source, culture history, or inoculum size influence the microbial growth. First, after seeding the cultures in LB broth from colony grown on LB agar plate, the *E. coli* BL21(DE3) and BW25113 cells were washed and transferred to M9 minimal medium containing either D-glucose or succinate. In the M9 minimal medium containing D-glucose, both the BL21(DE3) and BW25113 strains had similar specific growth rates (μ = 0.76 and 0.75 h^–1^, respectively) and reached the stationary phase within 24 h. However, in the case of the succinate medium, it took 48 h for the BL21(DE3) cells to reach maximum OD; moreover, they had a very long lag period (∼24 h). In contrast, the BW25113 cells showed a shorter lag phase (∼12 h) in the same medium (Figure 1A and Supplementary Figure 1A).

Second, we investigated whether the preculture medium affects the growth rate in the main culture. Seed cultures of the BW25113 or BL21(DE3) cells in M9 succinate broth and LB broth were inoculated in an M9 succinate medium. When the M9 succinate medium was used for the preculture, the lag period in the main culture was significantly reduced for both, compared to preculture in LB broth (Figure 1B and Supplementary Figure 1B). This result indicates that cells grown in preculture medium that is the same as the main culture medium grow faster.

Finally, we tested the effect of inoculum size on the lag period in the succinate medium. The lag period of the BW25113 cells was observed to be directly proportional to the serial dilution of the inoculated cells. However, for the BL21(DE3) cells, the intervals of each lag period between inoculum sizes were not sequential, and the lag time of smaller inoculum sizes (10^7^ and 10^0^ cells) was shorter than that of larger ones (10^8^ and 10^10^ cells) (Figure 1C and Supplementary Figure 1C). This phenomenon led us to study the BL21(DE3) cells more closely during the lag phase.

### Occurrence of Large Colonies During Transfer to Succinate Minimal Medium

**FIGURE 2:**
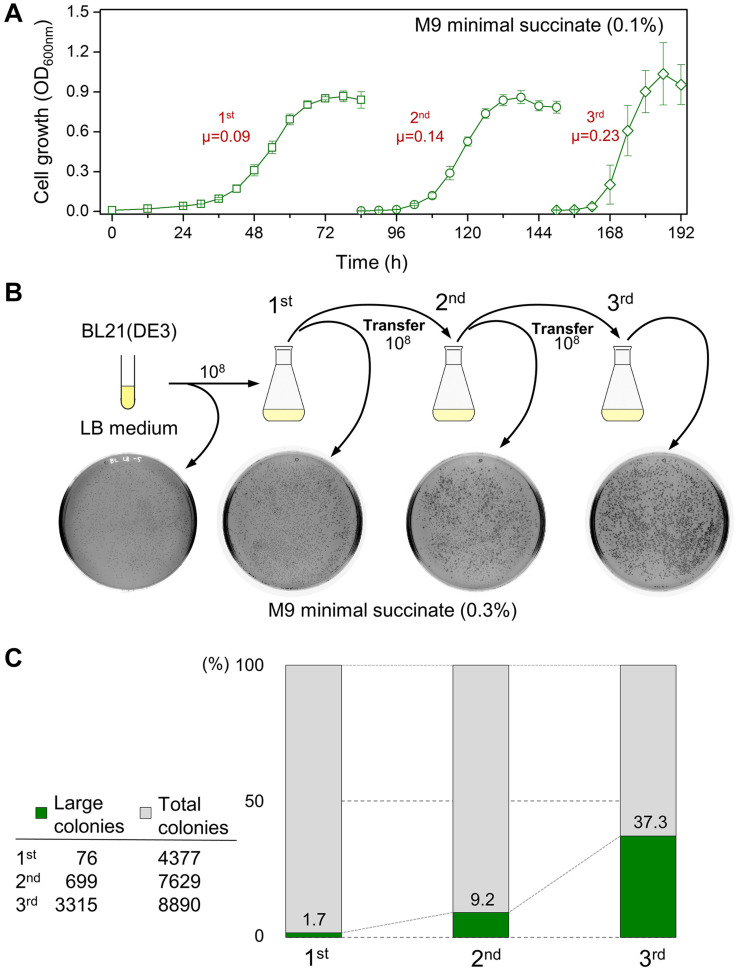
The growth profile of the BL21(DE3) cells during continuous transfer to fresh M9 succinate medium and the analysis of the size of colonies from the cultures. **(A)** Accelerated cell growth by serial seed transfer to fresh succinate medium. The unit of specific growth rate (μ) is h^– 1^. **(B)** The occurrence of large colonies and the increase in the number of large colonies from the cultures. **(C)** The ratio of the number of large to small colonies that grew after spread plating the culture samples.

We tested whether the serial transfer of the BL21(DE3) cells in minimal succinate medium could reduce the long lag time (Figure 2A and Supplementary Figure 2). Both the lag period and doubling time of the BL21(DE3) cells were shortened. When grown BL21(DE3) cells from each flask were spread on M9 agar containing succinate, a mixture of large (diameter ≥ 0.1 cm) and small (diameter ∼0.03 cm) colonies was observed (Figure 2B and Supplementary Figure 3). The proportion of large colonies growing on the agar plates compared to the small colonies increased dramatically with each transfer of inoculum to fresh succinate medium, accounting for 37.3% of the grown colonies in the third flask (Figure 2C).

Both large and small colonies were taken and streaked on MacConkey agar containing D-galactose. In parallel, the chromosomal T7 RNA polymerase gene was assessed via PCR. The results indicate that this was not the result of contamination with other bacterial cells such as BW25113 (Gal^+^); it was confirmed that both the large and small colonies were BL21(DE3) cells (Gal^–^). These results show that shortened lag periods and increased specific growth rates during adaptation might be due to a mixture of the large and small colony-forming BL21(DE3) cells.

### Genome and Transcript Analysis of Adapted HK565 Cells Derived From BL21(DE3)

**FIGURE 3:**
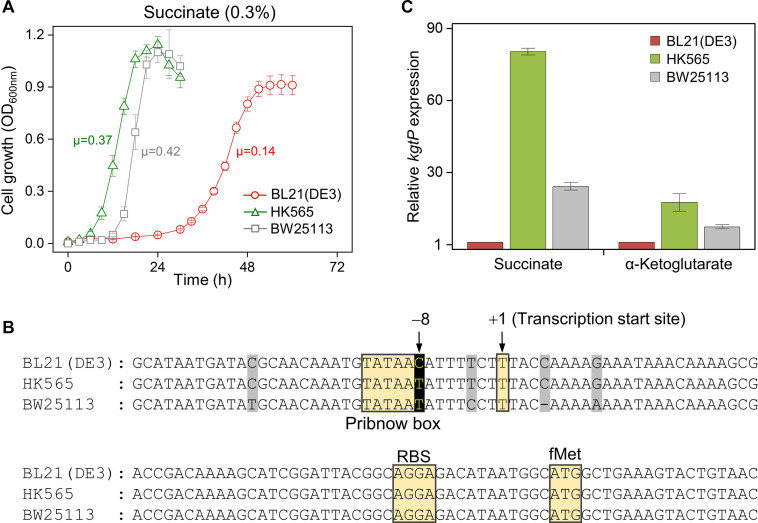
Characterization of the HK565 cells adapted in succinate minimal medium, compared to the BW25113 and parental BL21(DE3) strains. **(A)** Growth profiles of the HK565, BW25113, and BL21(DE3) strains. The unit of specific growth rate (μ) is h^– 1^. **(B)** Nucleotide sequence alignment of the promoter region of the *kgtP* gene in the HK565, BW25113, and BL21(DE3) strains. The nucleotides that evolved from C to T at the – 8 position of HK565 are shown in black. Unconserved nucleotide sequences are shaded in gray. **(C)** RT-qPCR analysis of the expression levels of *kgtP* in cells grown in M9 medium containing succinate or α-ketoglutarate.

Two different small and large colonies of BL21(DE3) on M9 minimal succinate agar were purified on LB agar plates and subsequently grown in the same M9 minimal succinate broth (Supplementary Figures 4, 5). While cells derived from the small colonies (*n* = 6) showed a long lag time (∼24 h) and slower growth (μ = 0.11 ± 0.01 h^–1^) like the original BL21(DE3) cells (μ = 0.14 h^–1^), cells derived from the large colonies (*n* = 20) showed a shortened lag phase (∼6 h) and an accelerated growth rate (μ = 0.36 ± 0.03 h^–1^). The HK565 cells, which were derived from large colonies, showed a shortened lag phase (∼6 h) and an accelerated growth rate (μ = 0.37 h^–1^), much like the BW25113 cells (μ = 0.42 h^–1^) (Figure 3A and Supplementary Figure 6).

Whole-genome sequencing of the HK565 cells resulted in the identification of a C-to-T single-nucleotide change that is located 73 nucleotides upstream from the AUG initiation codon of the *kgtP* gene, which encodes an α-ketoglutarate transporter, compared to the genome of the parental BL21(DE3) strain (Jeong et al., 2009). In addition, the 5′-end of the *kgtP* transcript was analyzed using 5′-RACE; the results showed that the C-to-T point mutation was located eight bases upstream from the transcription start site (+ 1). It was predicted that the –8C-to-T mutation corresponded to the consensus sequence of the Pribnow box (–10 sequence) of *kgtP* in the HK565 strain. The promoter sequence of *kgtP* in BW25113 is also shown (Figure 3B).

To check whether the –8C-to-T point mutation affects the transcription of *kgtP*, the mRNA expression level of *kgtP* was compared among the BL21(DE3), HK565, and BW25113 strains grown in M9 minimal medium containing either succinate or α-ketoglutarate (Figure 3C). The transcript of *kgtP* in HK565 was highly expressed in the presence of succinate (∼80-fold compared to the parental BL21(DE3)) or α-ketoglutarate (∼20-fold). This means that the parental BL21(DE3) strain has very little promoter activity for the *kgtP* gene and that a dicarboxylate-responsive strong promoter was created by the –8C-to-T point mutation in the adapted HK565 strain.

### Growth Rates Determined by Single-Nucleotide Changes in the Promoter of the *kgtP* Gene

The HK565 cells were grown in M9 minimal medium containing succinate or α-ketoglutarate as the sole carbon source. The HK565 cells showed a short lag time (<6 h) compared to the long lag time (∼24 h) of BL21(DE3) cells even if the preculture was grown in LB broth (Supplementary Figures 7, 8). Genetic complementation tests were performed via P1 phage transduction to confirm that the C-to-T mutation in the HK565 cells is responsible for the accelerated growth and eliminate the possibility that other mutations in the HK565 cells might be involved.

**FIGURE 4:**
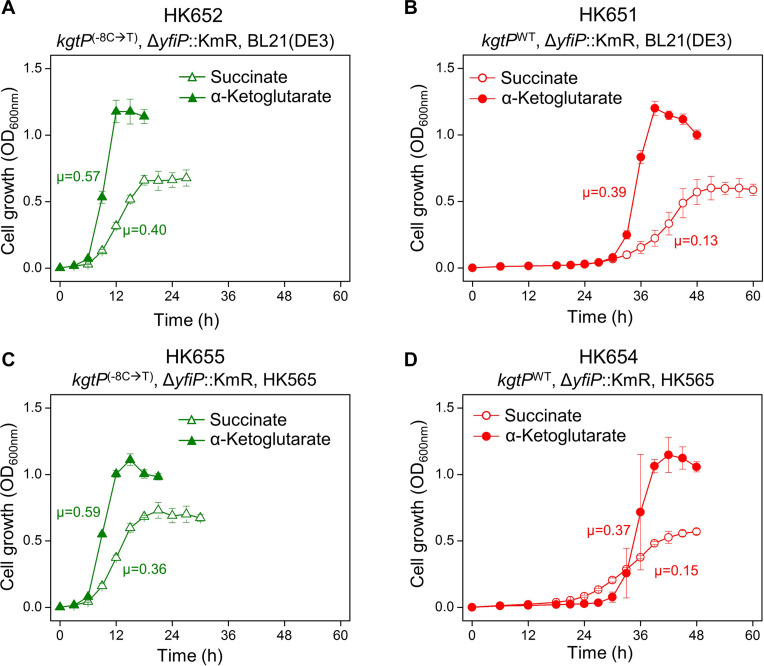
Effect of the – 8C-to-T single point mutation at the promoter of the *kgtP* gene on cellular growth in M9 minimal medium containing succinate or α-ketoglutarate. **(A)** *kgtP*^(–8C →T)^/BL21(DE3), **(B)** *kgtP*^WT^/BL21(DE3), **(C)** *kgtP*^(–8C →T)^/HK565, and **(D)** *kgtP*^WT^/HK565. *kgtP*^(– 8C →T)^ and *kgtP*^WT^ were transferred via P1 phage transduction to generate mutant cells from BL21(DE3) and revertant cells from the HK565 strain, respectively. The unit of specific growth rate (μ) is h^– 1^.

The single point –8T mutation carrying Δ*yfiP*::KmR was introduced into parental BL21(DE3) cells to generate the HK652 strain. When the HK652 cells, grown in LB broth, were washed and subsequently transferred to M9 broth containing succinate or α-ketoglutarate, cell growth was observed to occur immediately without a long lag phase, and the specific growth rate was increased (Figure 4A and Supplementary Figure 9A). When P1 lysates carrying –8C (Δ*yfiP*::KmR) were used to transduce the BL21(DE3) cells, the long lag period was not shortened (Figure 4B and Supplementary Figure 9B). When P1 lysates carrying –8T (Δ*yfiP*::KmR) were used to transduce the adapted HK565 cells, short lag phase was still observed (Figure 4C and Supplementary Figure 9C). However, when the –8T-to-C reverse mutation (back to wild type) was introduced into the HK565 cells, much slower growth with a long lag phase was observed again (Figure 4D and Supplementary Figure 9D). These results show that the –8C/T single-nucleotide change in *kgtP* in a BL21(DE3) background is a molecular switch for slow or quick cell growth in minimal medium containing succinate or α-ketoglutarate.

### Another Mode of Adaptation Revealed by the Δ*kgtP* Culture

To determine whether there is another way for the BL21(DE3) strain to adapt in M9 minimal succinate medium, we performed the same adaptation experiment with BL21(DE3) Δ*kgtP* cells. After a very long lag period (∼72 h) in the minimal succinate broth, we spread plated the Δ*kgtP* cells on minimal succinate agar, purified large colonies, and performed genome sequencing on one of the adapted Δ*kgtP* colonies. We identified a reverse mutation of the *dcuS* gene in the HK569 cells (Supplementary Figures 10A, 11), which is known to be a sensor kinase gene in a two-component system responsible for the utilization of four-carbon dicarboxylic acid (Worner et al., 2016).

The growth rates of the adapted HK565 (*kgtP*^(–8C →T)^) and HK569 (Δ*kgtP dcuS^rev^*) cells were compared. The specific growth rate of HK565 (*kgtP*^(–8C →T)^) and HK569 (Δ*kgtP dcuS^rev^*) in minimal succinate medium was calculated as 0.37 and 0.29 h^–1^, respectively (Supplementary Figures 10A, 11). This indicates that the HK565 cells carrying *kgtP*^(–8C →T)^ have higher fitness, compared to HK569 (Δ*kgtP dcuS^rev^*) cells in M9 minimal succinate broth. Moreover, Sanger DNA sequencing of 10 large colonies on minimal succinate agar from different cultures in the BL21(DE3) adaptation experiment showed only the –8C-to-T mutation in the *kgtP* gene, not the *dcuS* reverse mutation in the genome. This result shows that the 5-bp deletion in the *dcuS* gene is more rarely obtained than the –8T-to-C single base mutation in the *kgtP* gene during the adaptation of the BL21(DE3) cells in M9 succinate medium.

### Visualization of Genomic Mutagenesis During Adaptation

To monitor the outbreak of the –8C-to-T single point mutation in the BL21(DE3) population in M9 minimal succinate medium, we designed translational fusion of the *kgtP* gene with the *sfgfp* gene in the genome (Supplementary Figure 12A). The insertion of the Gly-Gly-Gly-Gly-Ser linker was designed between the C-terminal of KgtP and the N-terminal of Sf-GFP. Finally, we constructed HK1020 (*kgtP*^WT^∼*sfgfp*) and HK1022 (*kgtP*^(–8C →T)^∼*sfgfp*), derived from the BL21(DE3) and HK565 cells, respectively. The growth rates of the HK1020 and HK1022 cells were not significantly different from the respective parental BL21(DE3) and HK565 cells, indicating that the C-terminal translational fusion does not affect the function of KgtP (Supplementary Figures 12B, 13). Moreover, we observed plenty of green fluorescence in the HK1022 cells and slight autofluorescence in the HK1020 cells, both in the stationary phase (Supplementary Figure 12C). These results indicate that the sfGFP reporter system can be used to monitor the promoter activity of *kgtP* in BL21(DE3).

**FIGURE 5:**
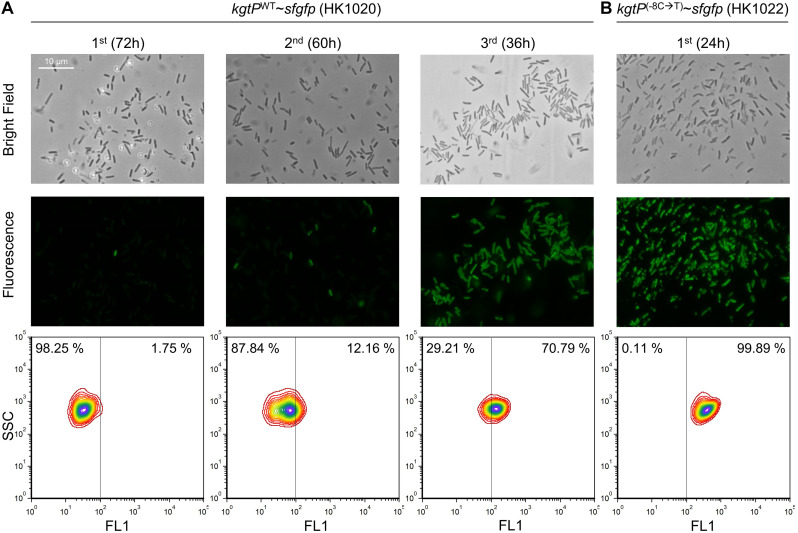
Fluorescence analysis of the HK1020 (*kgtP*^WT^∼*sfgfp*) and HK1022 (*kgtP*^(–8C →T)^∼*sfgfp*) cells grown in M9 succinate medium. The occurrence and the number of fluorescent cells in the HK1020 population were analyzed via microscopy (top: bright field, middle: fluorescence) and flow cytometry (bottom), respectively.

Before the adaptation experiment of the HK1020 cells, we evaluated the fluorescence expression in the HK1022 (*kgtP*^(–8C →T)^∼*sfgfp*) cells. HK1022 cells grown in minimal succinate broth showed strong fluorescence that could be observed clearly under the fluorescence microscope and the flow cytometer. Next, we performed the serial transfer experiment with the HK1020 (*kgtP*^WT^∼*sfgfp*) cells that were derived from BL21(DE3) (Figure 5). After the first round of flask culture, several fluorescent cells were observed among the hundreds of cells under the fluorescence microscope. The number of fluorescent cells was quantitatively measured via flow cytometry. Of the total cells, 1.75% (17,500/1,000,000) of cells showed significant fluorescence. During the second round, the lag time of the HK1020 cells was shortened (∼12 h), similar to the adaptation of BL21(DE3), and the number of fluorescent cells increased to 12.16%. Lastly, more than half of the cells (70.79%) were found to be fluorescent at the end of the third culture, and –8C-to-T mutations in the *kgtP* gene of 10 fluorescent colonies were confirmed via Sanger sequencing. These results show that fluorescent cells carrying *kgtP*^(–8C →T)^ emerge immediately in the minimal succinate environment and that mutant cells dominate the microbial population in a short period.

## Discussion

The study of the microbial lag phase is considered to be in line with the question of how microbes adapt to the environment, as even the same bacterial cells have different lag times depending on the culture conditions, culture history, and seed size (Dean, 1957; Robinson et al., 2001; Vermeersch et al., 2019). As the prolonged lag phase of the BL21 cells has been observed in M9 minimal succinate medium (Paliy and Gunasekera, 2007), we used BL21(DE3), a derivative of the BL21 strain, as a model system to study bacterial adaptation to minimal succinate medium. We observed that media differences in preculture can produce dramatically different lag lengths without significant changes in exponential growth, which implies mostly physiological adaptation in the lag phase (Figure 1B). Our inoculum dilution experiment yielded results similar to those of a previous study (Paliy and Gunasekera, 2007), but the lag time of the BL21(DE3) cells was reversed at cell counts of 10^7^ and 10^8^ (Figure 1C). This led to the idea that not all cells in a bacterial population are physiologically homogenous.

If the lag time is simply the preparation time of microorganisms for a new environment, only the lag time should be reduced when serial transfer is performed; however, a gradual increase in the specific growth rates was also observed (Figure 2A), implying that the rate of physiological metabolism increased. We observed a mixture of small and large colonies taken from the culture of BL21(DE3) (Figures 2B,C); we found that the lag time of the cells from the large colonies was shortened, and the cells grew rapidly in minimal succinate medium (Supplementary Figures 4, 5). As the length of the lag phase and the specific growth rate are expressed as average values of all cells in the population, the increase in the number of large colonies appears to be related to faster growth rates in the second and third cultures. Therefore, we considered this phenomenon to be a result of acquired microbial traits and not a transient phenomenon and performed genome analysis to find the answer.

Whole-genome sequencing showed a single-nucleotide C-to-T change in the promoter of the *kgtP* gene, which encodes an α-ketoglutarate transporter (Seol and Shatkin, 1991), in the genome of adapted BL21(DE3) cells, which is caused by spontaneous base mutations at a rate of 8.9 × 10^–10^ per base per generation (Wielgoss et al., 2011). This mutation appeared to create the consensus –10 sequence in the promoter and activate *kgtP* (Figures 3B,C). Cells carrying the C-to-T mutation may or may not already exist in the seed. However, the probability that the mutation originally existed increases with the seed size. The mutation may also arise newly during transfer. The genetic heterogeneity of microbial populations caused by spontaneous mutations enables mutant cells with better fitness to rapidly adapt and form subpopulations in changed or restricted environments.

A genetic complementation test confirmed that the single mutation is responsible for the shortened lag phase and rapid growth of the genetically adapted BL21(DE3) cells in minimal succinate medium (Figure 4). It is known that *kgtP* is responsible for growth on α-ketoglutarate (Seol and Shatkin, 1991) and that KgtP-mediated α-ketoglutarate transport could be partially inhibited by succinate (Seol and Shatkin, 1992). Our study showed that KgtP can also play a role in the efficient uptake of succinate, as well as α-ketoglutarate (Figure 4).

To confirm whether there is another method by which BL21(DE3) adapts in succinate medium, we grew the BL21(DE3) Δ*kgtP* cells for 144 h to obtain the HK569 (Δ*kgtP dcuS^rev^*) cells (Supplementary Figures 10, 11). It was observed that the specific growth rate of the adapted HK569 cells was lower when compared with HK565 (*kgtP*^(–8C →T)^). This is probably why we could not find cells with the *dcuS*^rev^ mutation among the adapted BL21(DE3) cells.

A fluorescent reporter gene was translationally fused to *kgtP* (Supplementary Figure 12), and the fluorescence of the sfGFP-KgtP fusion protein was monitored to determine how cells having C-to-T mutations in the promoter of the *kgtP* gene occupy the population when BL21(DE3) cells are grown in succinate minimal medium. When transferred once, twice, and thrice in succinate minimal medium, C-to-T mutation–containing cells accounted for 1.75, 12.16, and 70.79%, respectively, of the microbial population (Figure 5). These results show the frequency of the said mutation in BL21(DE3) cells during adaptation to the minimal succinate medium, as well as the adaptation process where the adapted cells carrying the mutation dominate the population.

In summary, microorganisms express the necessary genes and optimize metabolism in the new culture medium, the length of which is surely influenced by culture history. In addition to this physiological adaptation, our study showed that the appearance of adapted cells in preculture by natural mutation can also contribute to accelerating cell growth and shortening the lag phase in the main culture. The genetic heterogeneity of microbial populations provides a variety of adaptability options for microbes to adapt to the changing natural environment, which may be one of the mechanisms by which microbes can adapt and survive in diverse environments in nature.

## Data Availability Statement

The datasets presented in this study can be found in online repositories. The names of the repository/repositories and accession number(s) can be found below: NCBI (accession: PRJNA529313).

## Author Contributions

HK and SL conceived the research, and wrote the main manuscript text. HK performed experiments. HJ analyzed genome sequencing data. All authors reviewed the manuscript.

## Conflict of Interest

The authors declare that the research was conducted in the absence of any commercial or financial relationships that could be construed as a potential conflict of interest.

## Footnotes

1 http://lifescience.roche.com

## Supplementary Material

Supplementary Figure 1 The logarithmic scale of the cell growth of *E. coli* BW25113 and BL21(DE3). **(A)** Different carbon sources; cells grown in LB broth were washed and transferred to M9 minimal medium containing D-glucose or succinate. **(B)** Different preculture medium; cells grown in LB and M9 succinate medium were washed and transferred to M9 succinate medium. **(C)** Different inoculum size; cells grown in LB broth were washed and transferred to M9 succinate medium. The unit of specific growth rate (μ) is h^–1^.

Supplementary Figure 2 The logarithmic scale of the growth profile of the BL21(DE3) cells during continuous transfer to fresh M9 succinate medium and the analysis of the size of colonies from the cultures. The unit of specific growth rate (μ) is h^–1^.

Supplementary Figure 3 Continuous transfer of BL21(DE3) cells to fresh M9 succinate medium **(A)** and the analysis of the size of colonies from the cultures **(B)**.

Supplementary Figure 4 Cell growth of colonies isolated from M9 succinate minimal agar after culture M9 minimal medium. **(A)** Small colonies (*n* = 6) and **(B)** Large colonies (*n* = 20). **(C)** Statistical comparison of specific growth rates (μ; h^–1^) of small and large colonies.

Supplementary Figure 5 The logarithmic scale of the cell growth of colonies isolated from M9 succinate minimal agar after culture M9 minimal medium. **(A)** Small colonies (*n* = 6) and **(B)** Large colonies (*n* = 20).

Supplementary Figure 6 The logarithmic scale of the growth profiles of the HK565, BW25113, and BL21(DE3) strains. The unit of specific growth rate (μ) is h^–1^.

Supplementary Figure 7 Comparison of the growth profiles of parental BL21(DE3) and adapted HK565 grown in minimal medium containing different carbon sources. **(A)** Succinate, **(B)** α-ketoglutarate, **(C)** D-glucose, and **(D)** glycerol. The unit of specific growth rate (μ) is h^–1^.

Supplementary Figure 8 The logarithmic scale of the growth profiles of parental BL21(DE3) and adapted HK565 grown in minimal medium containing different carbon sources. **(A)** Succinate, **(B)** α-ketoglutarate, **(C)** D-glucose, and **(D)** glycerol. The unit of specific growth rate (μ) is h^–1^.

Supplementary Figure 9 The logarithmic scale of the cell growth profiles of cells carrying − 8C-to-T single point mutation at the promoter of the *kgtP* gene. **(A)** *kgtP*^(–8C →T)^/BL21(DE3), **(B)** *kgtP*^WT^/BL21(DE3), **(C)** *kgtP*^(–8C →T)^/HK565, and **(D)** *kgtP*^WT^/HK565, *kgtP*^(–8C →T)^, and *kgtP*^WT^ were transferred via P1 phage transduction to generate mutant cells from BL21(DE3) and revertant cells from the HK565 strain, respectively. The unit of specific growth rate (μ) is h^–1^.

Supplementary Figure 10 Adaptation of the Δ*kgtP* cells (HK567) in M9 succinate minimal medium. **(A)** Growth profiles of parental Δ*kgtP* cells (HK567) and adapted Δ*kgtP* cells (HK569). The unit of specific growth rate (μ) is h^–1^. **(B)** Deletion of CGCCG (5 bp) in the middle of the open reading frame of the *dcuS* gene in the adapted Δ*kgtP* cells (HK569). In the parental BL21(DE3) strain, the gene product of the *dcuS* gene appears to be prematurely terminated due to the frameshift caused by three repeats of CGCCG.

Supplementary Figure 11 The logarithmic scale of the growth profiles of parental Δ*kgtP* cells (HK567) and adapted Δ*kgtP* cells (HK569). The unit of specific growth rate (μ) is h^–1^.

Supplementary Figure 12 The translational fusion of the *kgtP* gene and *sfgfp* gene for the monitoring of the − 8C-to-T mutation at the promoter of the *kgtP* gene. **(A)** Genomic construction of *kgtP*∼*sfgfp* in BL21(DE3) and BK565 to generate the HK1020 and HK1022 cells, respectively. **(B)** Comparison of the growth profiles of cells carrying the *kgtP* gene alone (BL21(DE3) and HK565) or *kgtP*∼*sfgfp* (HK1020 and HK1022) in the genome. **(C)** Analysis of fluorescence intensity in the cultures of the HK1020 and HK1022 strains.

Supplementary Figure 13 Comparison of the logarithmic scale of the growth profiles of cells carrying the *kgtP* gene alone (BL21(DE3) and HK565) or *kgtP*∼*sfgfp* (HK1020 and HK1022) in the genome.

Supplementary Table 1 Primers used in this study.

Supplementary Datasheet Cell growth data on a logarithmic scale of the strains in this study.
