# Supplementary material for: Visualization and Quantification of Genetically Adapted Microbial Cells During Preculture
Source: Front Microbiol. 2021 Jul 14;12:693464. doi: 10.3389/fmicb.2021.693464 (PMC8317463; doi:10.3389/fmicb.2021.693464)
Supplement: Supplementary Figure 1 — The logarithmic scale of the cell growth of E. coli BW25113 and BL21(DE3). (A) Different carbon sources; cells grown in LB broth were washed and transferred to M9 minimal medium containing D-glucose or succinate. (B) Different preculture medium; cells grown in LB and M9 succinate medium were washed and transferred to M9 succinate medium. (C) Different inoculum size; cells grown in LB broth were washed and transferred to M9 succinate medium. The unit of specific growth rate (μ) is h–1. [file Presentation_1.PPTX]

## Slide 1
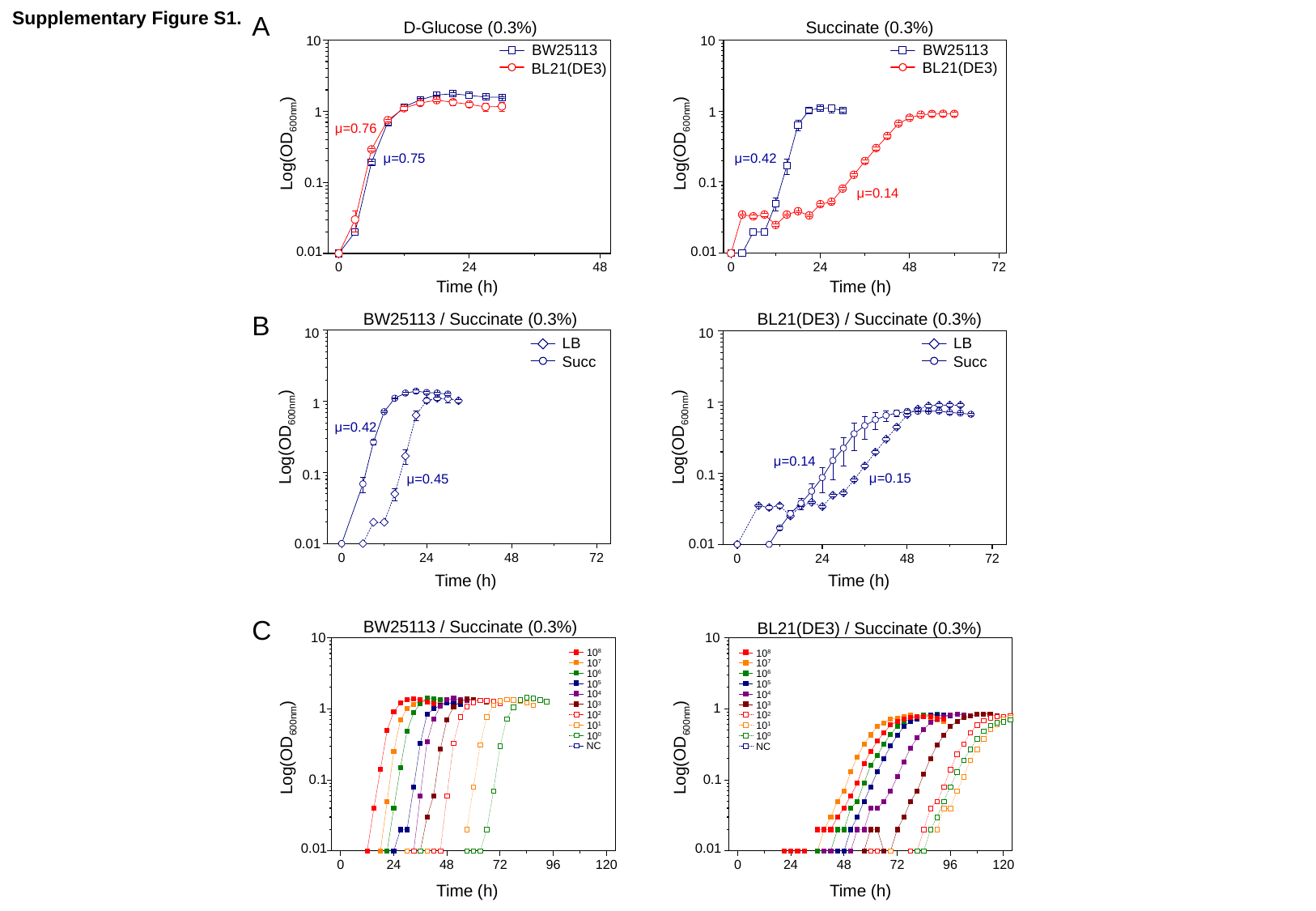

Supplementary Figure S1.
A
Succinate (0.3%)
D-Glucose (0.3%)
10
1
0.1
0.01
10
1
0.1
0.01
BW25113
BL21(DE3)
BW25113
BL21(DE3)
μ=0.76
Log(OD600nm)
Log(OD600nm)
μ=0.75
μ=0.42
μ=0.14
Time (h)
Time (h)
B
BW25113 / Succinate (0.3%)
BL21(DE3) / Succinate (0.3%)
10
1
0.1
0.01
10
1
0.1
0.01
LB
Succ
LB
Succ
μ=0.42
Log(OD600nm)
Log(OD600nm)
μ=0.14
μ=0.15
μ=0.45
Time (h)
Time (h)
C
BW25113 / Succinate (0.3%)
BL21(DE3) / Succinate (0.3%)
10
1
0.1
0.01
10
1
0.1
0.01
108
107
106
105
104
103
102
101
100
NC
108
107
106
105
104
103
102
101
100
NC
Log(OD600nm)
Log(OD600nm)
Time (h)
Time (h)

## Slide 2
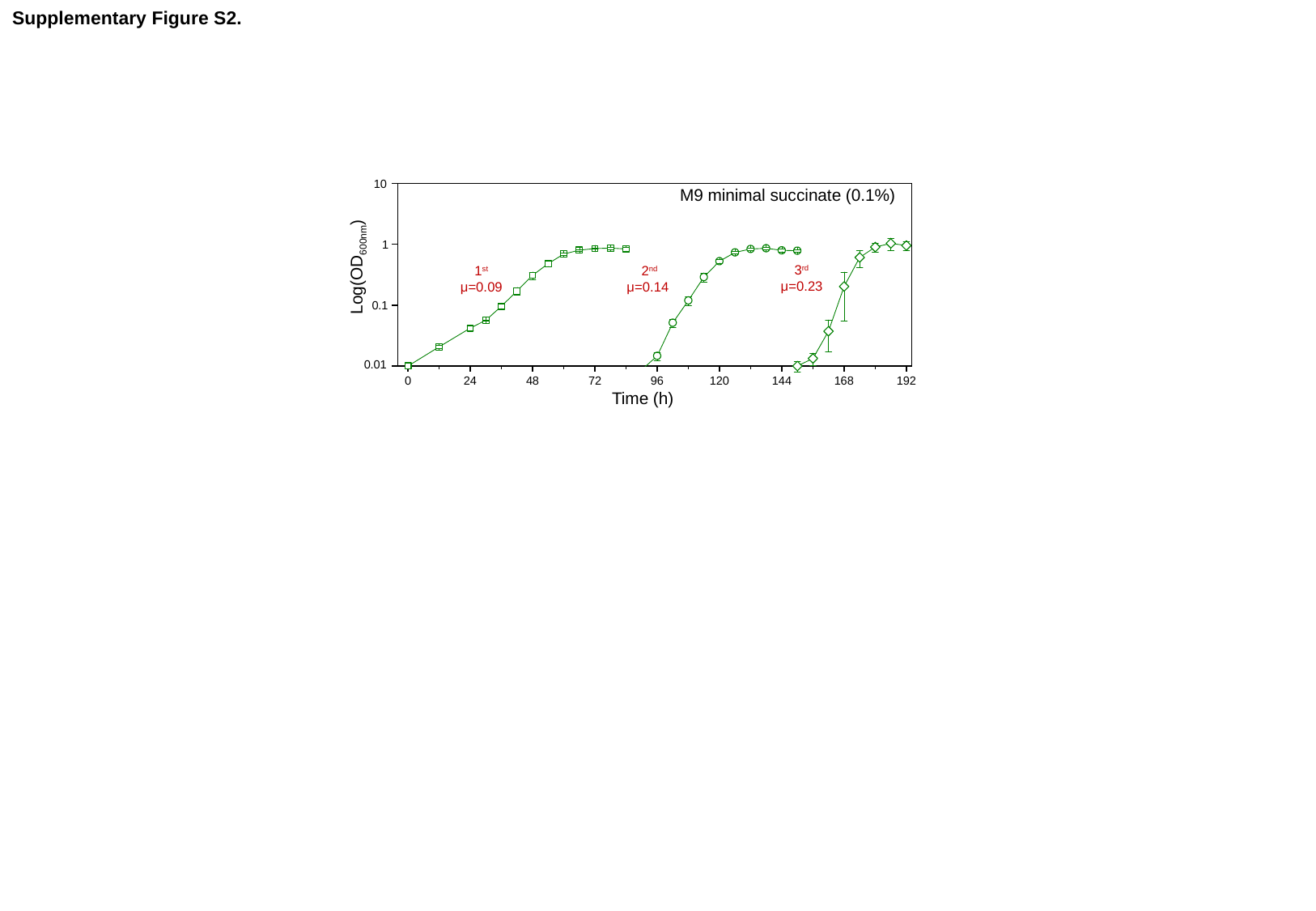

Supplementary Figure S2.
10
1
0.1
0.01
M9 minimal succinate (0.1%)
Log(OD600nm)
3rd
μ=0.23
1st
μ=0.09
2nd
μ=0.14
Time (h)

## Slide 3
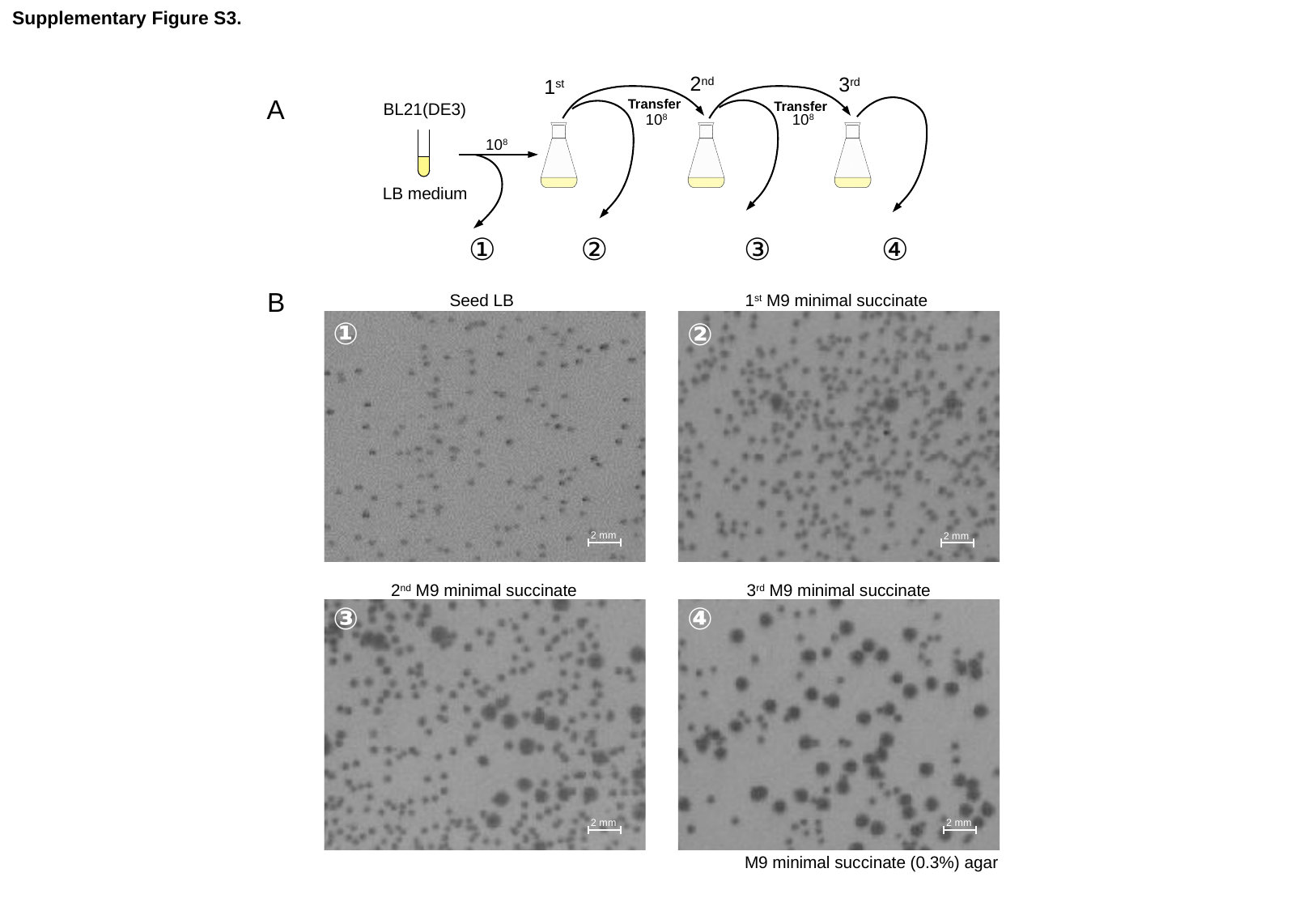

Supplementary Figure S3.
2nd
3rd
1st
Transfer
Transfer
108
108
A
BL21(DE3)
108
LB medium
① ② ③ ④
B
Seed LB
1st M9 minimal succinate
①
②
2 mm
2 mm
2nd M9 minimal succinate
3rd M9 minimal succinate
③
④
2 mm
2 mm
M9 minimal succinate (0.3%) agar

## Slide 4
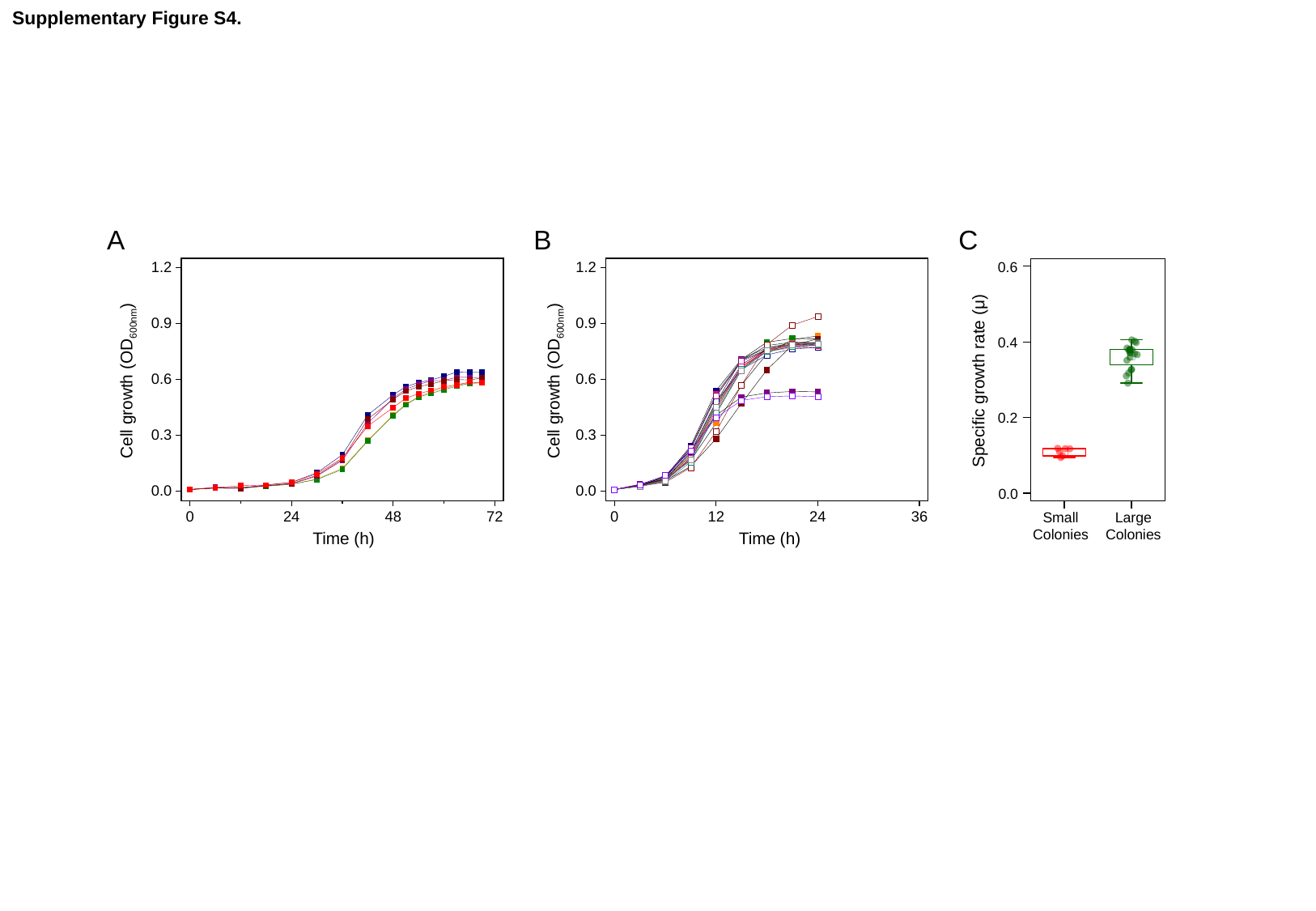

Supplementary Figure S4.
A
B
C
0.6
0.4
Specific growth rate (μ)
0.2
0.0
Small
Colonies
Large
Colonies
Cell growth (OD600nm)
Cell growth (OD600nm)
Time (h)
Time (h)

## Slide 5
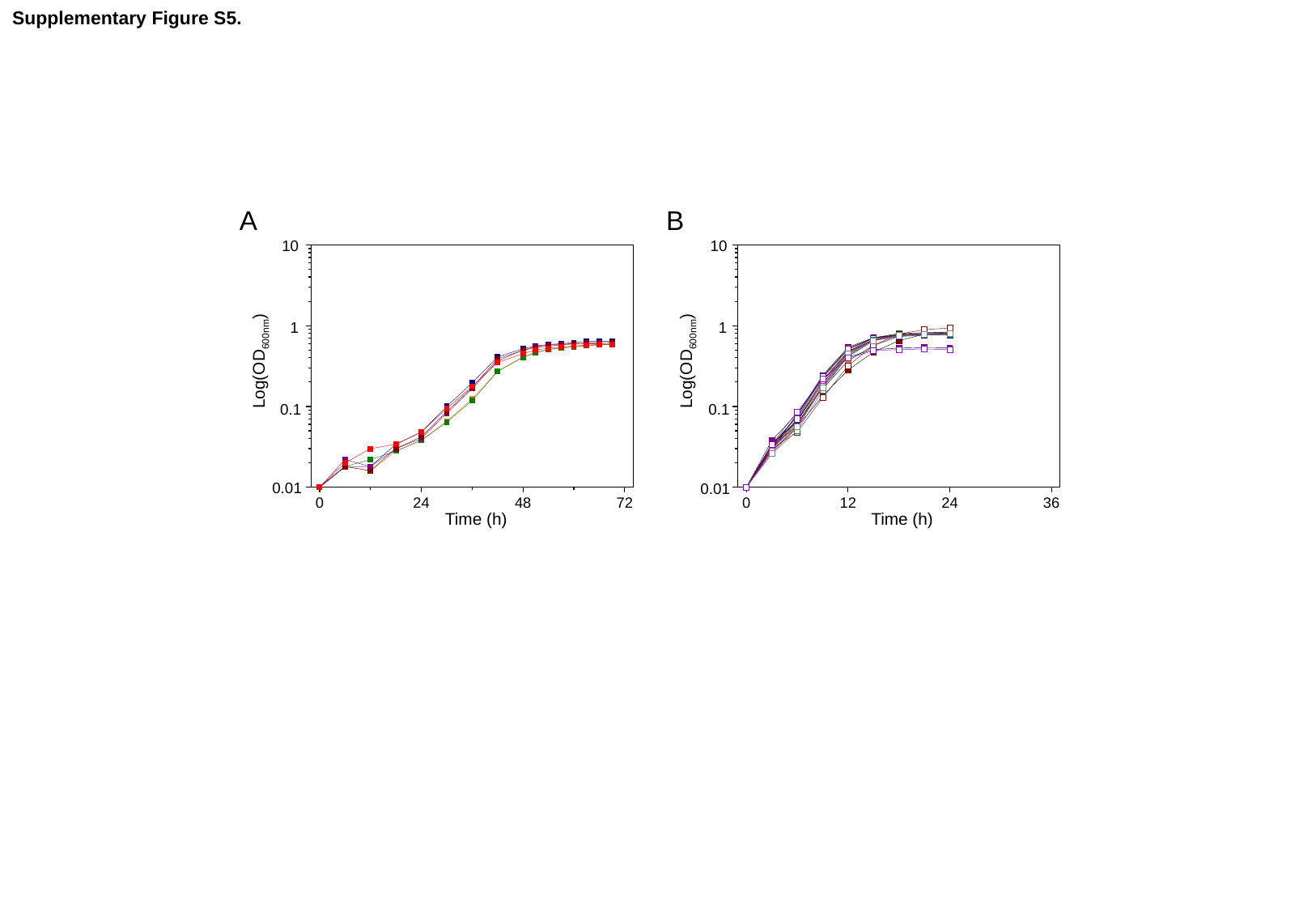

Supplementary Figure S5.
A
B
10
1
0.1
0.01
10
1
0.1
0.01
Log(OD600nm)
Log(OD600nm)
Time (h)
Time (h)

## Slide 6
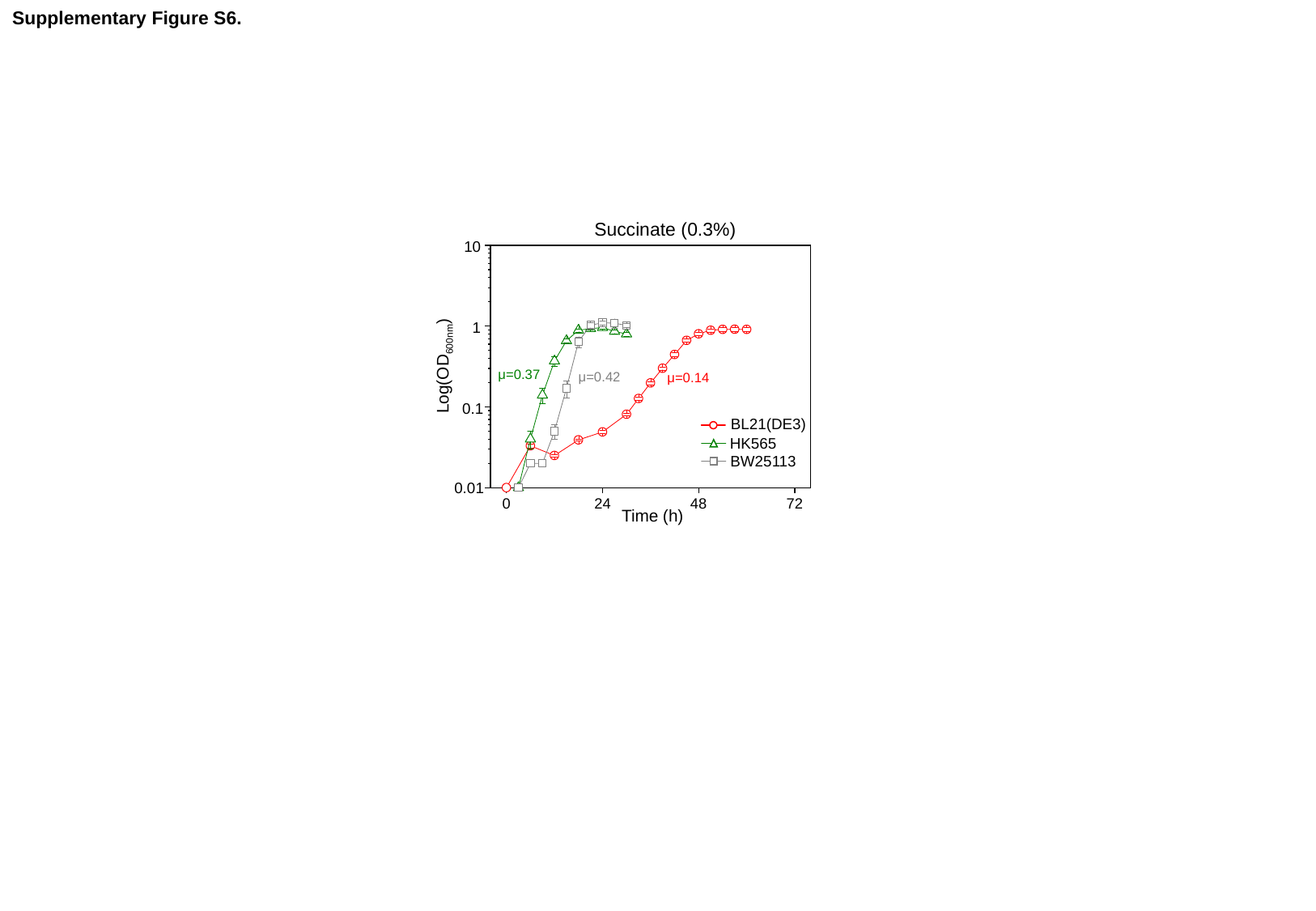

Supplementary Figure S6.
Succinate (0.3%)
10
1
0.1
0.01
Log(OD600nm)
Time (h)
μ=0.37
μ=0.42
μ=0.14
BL21(DE3)
HK565
BW25113

## Slide 7
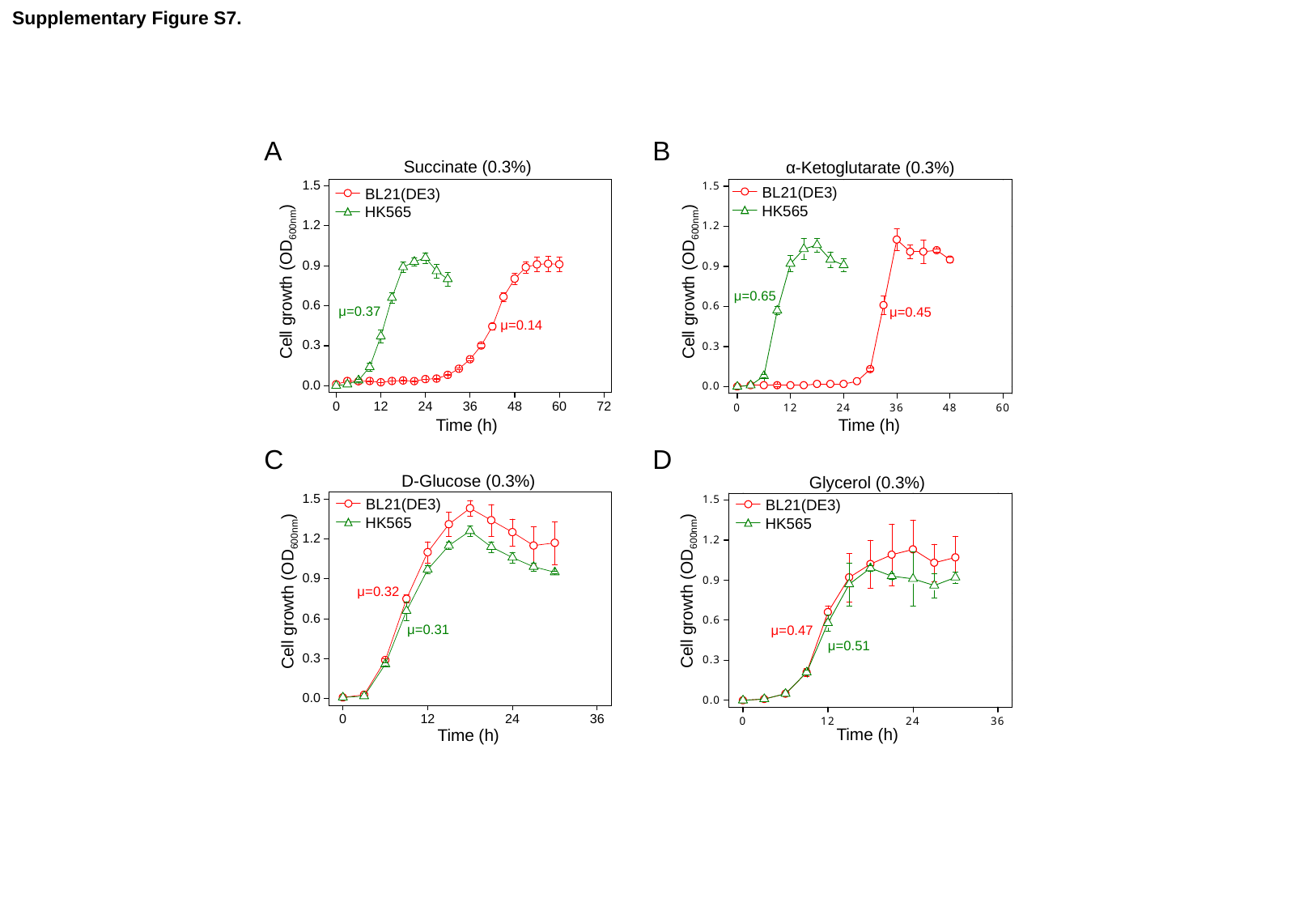

Supplementary Figure S7.
A
B
Succinate (0.3%)
BL21(DE3)
HK565
Cell growth (OD600nm)
Time (h)
α-Ketoglutarate (0.3%)
BL21(DE3)
HK565
Cell growth (OD600nm)
Time (h)
μ=0.65
μ=0.37
μ=0.45
μ=0.14
C
D
D-Glucose (0.3%)
BL21(DE3)
HK565
Cell growth (OD600nm)
Time (h)
Glycerol (0.3%)
BL21(DE3)
HK565
Cell growth (OD600nm)
Time (h)
μ=0.32
μ=0.31
μ=0.47
μ=0.51

## Slide 8
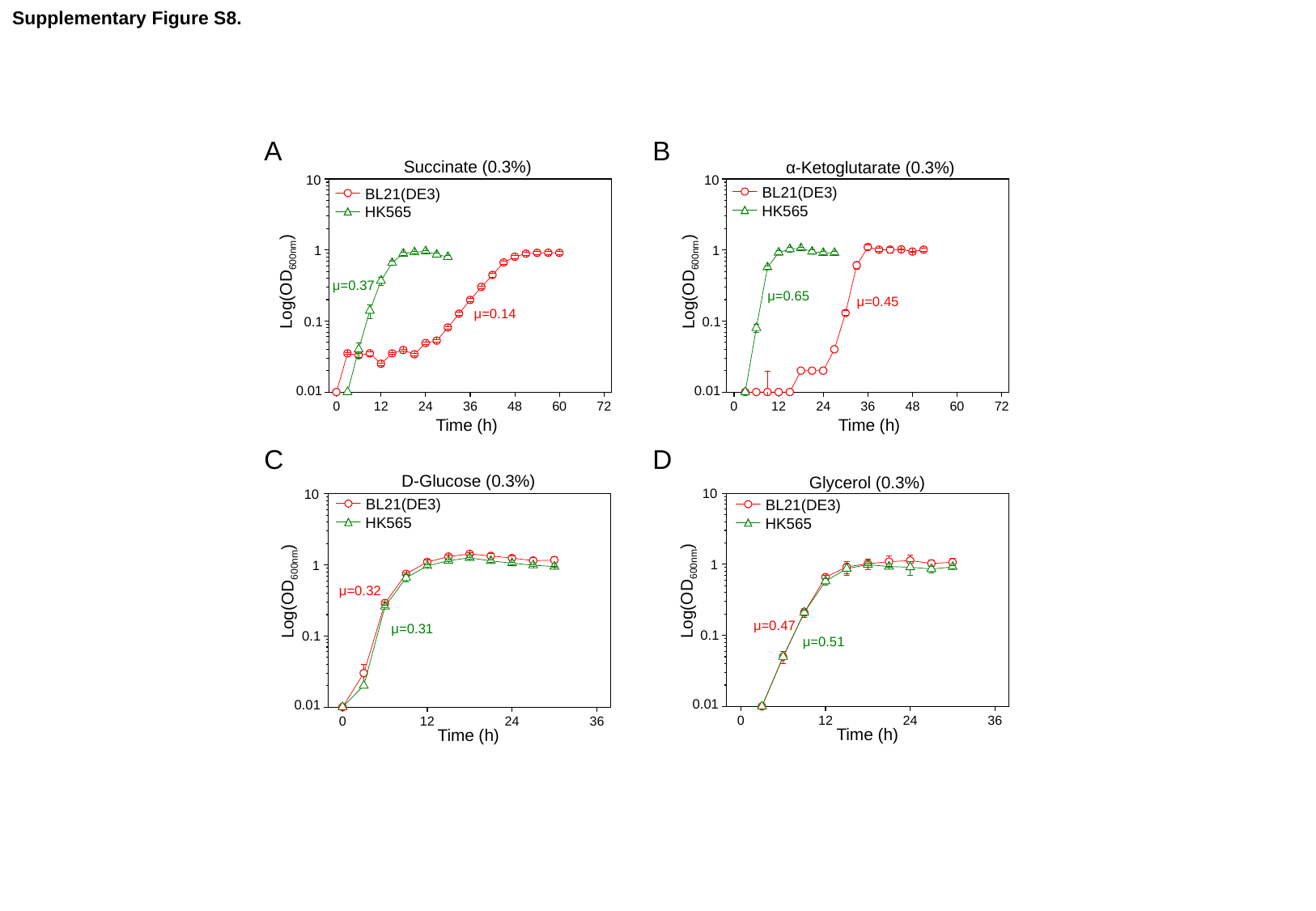

Supplementary Figure S8.
A
B
Succinate (0.3%)
BL21(DE3)
HK565
Log(OD600nm)
Time (h)
α-Ketoglutarate (0.3%)
BL21(DE3)
HK565
Log(OD600nm)
Time (h)
10
1
0.1
0.01
10
1
0.1
0.01
μ=0.37
μ=0.65
μ=0.45
μ=0.14
C
D
D-Glucose (0.3%)
BL21(DE3)
HK565
Log(OD600nm)
Time (h)
Glycerol (0.3%)
BL21(DE3)
HK565
Log(OD600nm)
Time (h)
10
1
0.1
0.01
10
1
0.1
0.01
μ=0.32
μ=0.47
μ=0.31
μ=0.51

## Slide 9
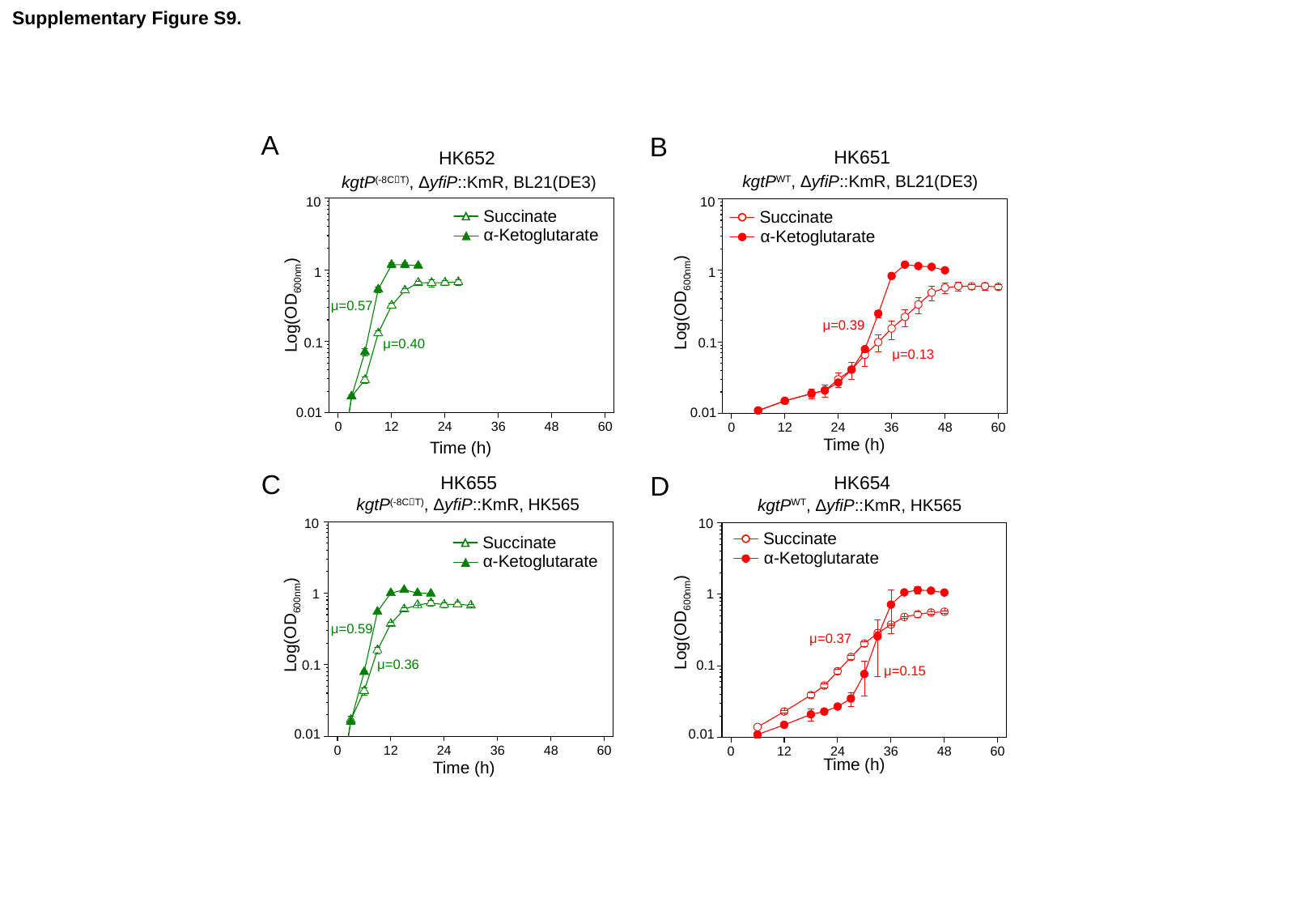

Supplementary Figure S9.
A
B
HK651
HK652
kgtPWT, ΔyfiP::KmR, BL21(DE3)
Succinate
α-Ketoglutarate
Log(OD600nm)
Time (h)
kgtPWT, ΔyfiP::KmR, HK565
Succinate
α-Ketoglutarate
Log(OD600nm)
Time (h)
kgtP(-­­­­8CT), ΔyfiP::KmR, BL21(DE3)
Succinate
α-Ketoglutarate
Log(OD600nm)
Time (h)
kgtP(-­­­­8CT), ΔyfiP::KmR, HK565
Succinate
α-Ketoglutarate
Log(OD600nm)
Time (h)
10
1
0.1
0.01
10
1
0.1
0.01
μ=0.57
μ=0.39
μ=0.40
μ=0.13
C
D
HK655
HK654
10
1
0.1
0.01
10
1
0.1
0.01
μ=0.59
μ=0.37
μ=0.36
μ=0.15

## Slide 10
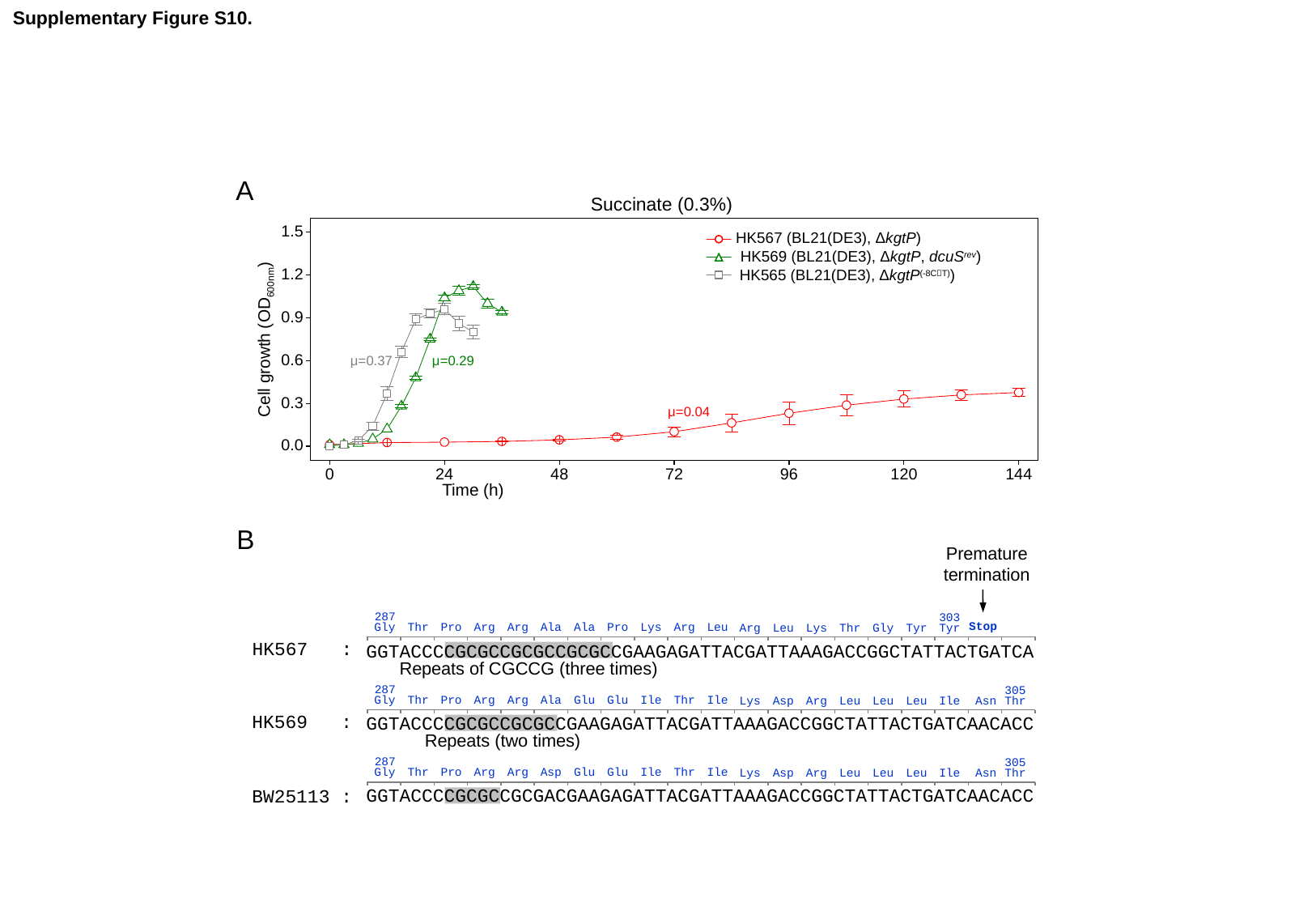

Supplementary Figure S10.
A
Succinate (0.3%)
HK567 (BL21(DE3), ΔkgtP)
HK569 (BL21(DE3), ΔkgtP, dcuSrev)
HK565 (BL21(DE3), ΔkgtP(-8CT))
Cell growth (OD600nm)
Time (h)
μ=0.37
μ=0.29
μ=0.04
B
Premature termination
287
303
Stop
Gly
Thr
Pro
Arg
Arg
Ala
Ala
Pro
Lys
Arg
Leu
Arg
Leu
Lys
Thr
Gly
Tyr
Tyr
GGTACCCCGCGCCGCGCCGCGCCGAAGAGATTACGATTAAAGACCGGCTATTACTGATCA
HK567 :
Repeats of CGCCG (three times)
287
305
Gly
Thr
Pro
Arg
Arg
Ala
Glu
Glu
Ile
Thr
Ile
Lys
Asp
Arg
Leu
Leu
Leu
Ile
Thr
Asn
GGTACCCCGCGCCGCGCCGAAGAGATTACGATTAAAGACCGGCTATTACTGATCAACACC
HK569 :
Repeats (two times)
287
305
Gly
Thr
Pro
Arg
Arg
Asp
Glu
Glu
Ile
Thr
Ile
Lys
Asp
Arg
Leu
Leu
Leu
Ile
Thr
Asn
GGTACCCCGCGCCGCGACGAAGAGATTACGATTAAAGACCGGCTATTACTGATCAACACC
BW25113 :

## Slide 11
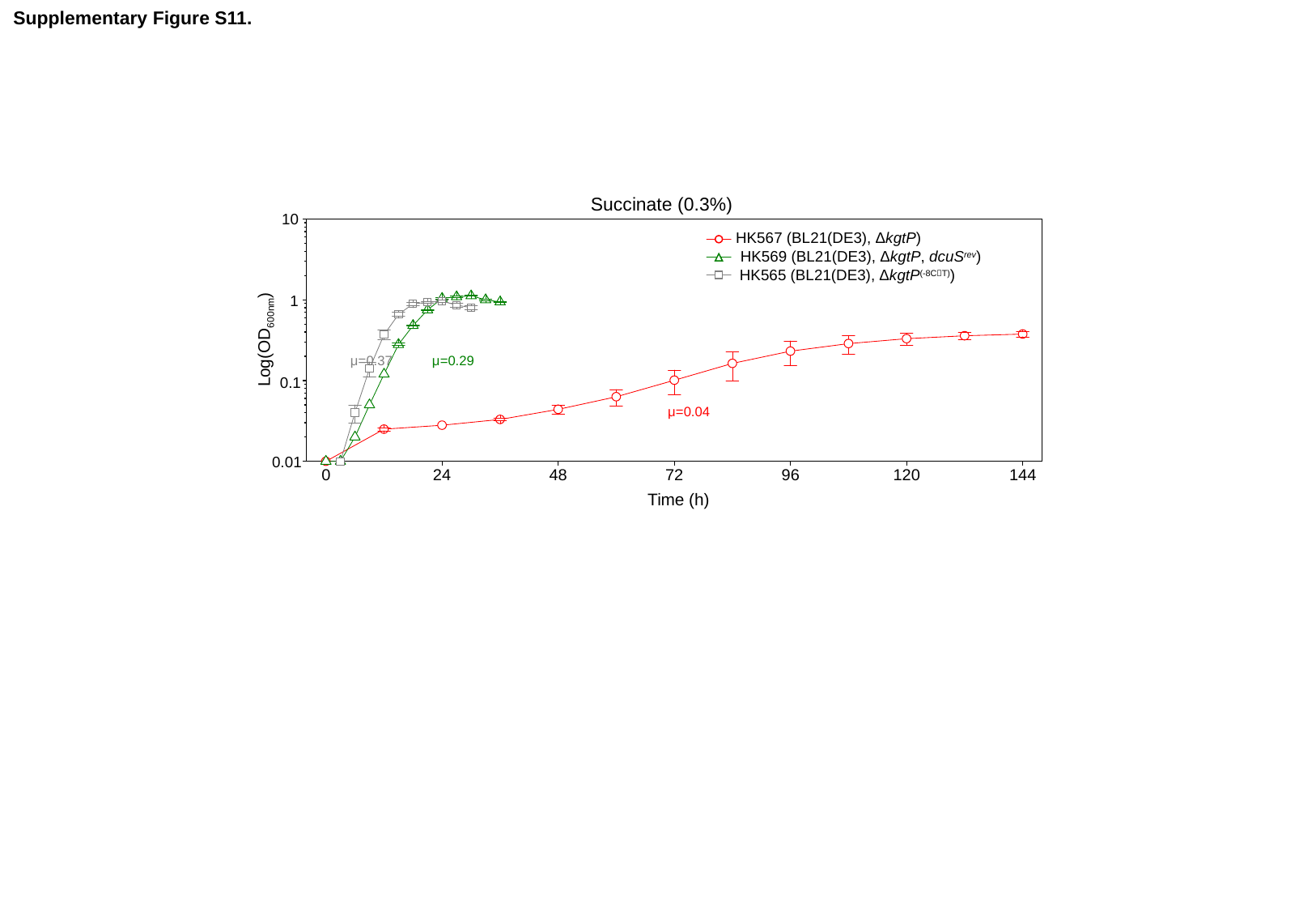

Supplementary Figure S11.
Succinate (0.3%)
10
1
0.1
0.01
HK567 (BL21(DE3), ΔkgtP)
HK569 (BL21(DE3), ΔkgtP, dcuSrev)
HK565 (BL21(DE3), ΔkgtP(-8CT))
Log(OD600nm)
Time (h)
μ=0.37
μ=0.29
μ=0.04

## Slide 12
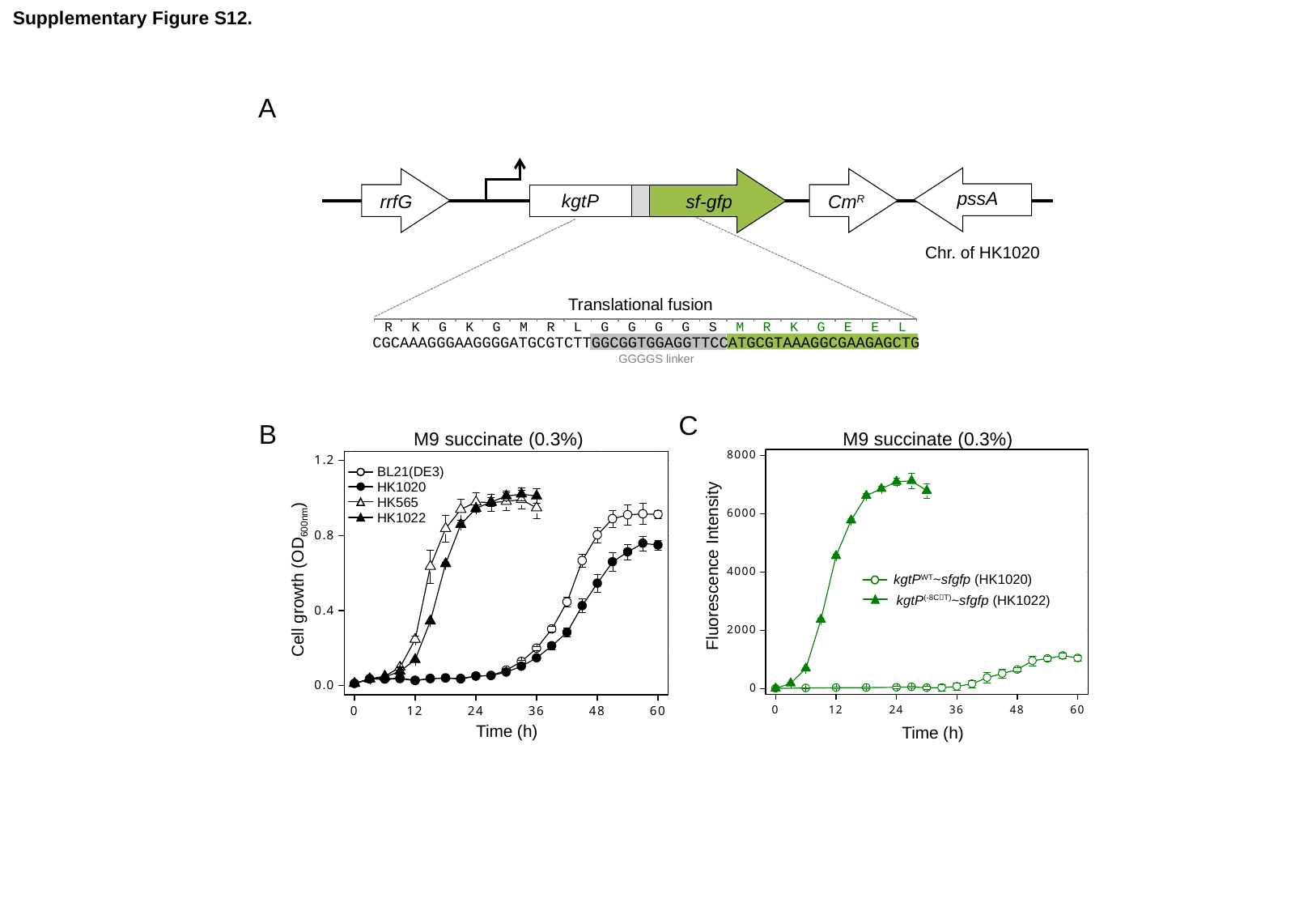

Supplementary Figure S12.
A
kgtP
sf-gfp
kgtP
pssA
rrfG
CmR
Chr. of HK1020
Translational fusion
G
S
M
R
K
G
E
E
L
R
K
G
K
G
M
R
L
G
G
G
CGCAAAGGGAAGGGGATGCGTCTTGGCGGTGGAGGTTCCATGCGTAAAGGCGAAGAGCTG
GGGGS linker
C
B
M9 succinate (0.3%)
M9 succinate (0.3%)
BL21(DE3)
HK1020
HK565
HK1022
Fluorescence Intensity
kgtPWT~sfgfp (HK1020)
kgtP(-­­­­8CT)~sfgfp (HK1022)
Time (h)
Cell growth (OD600nm)
Time (h)

## Slide 13
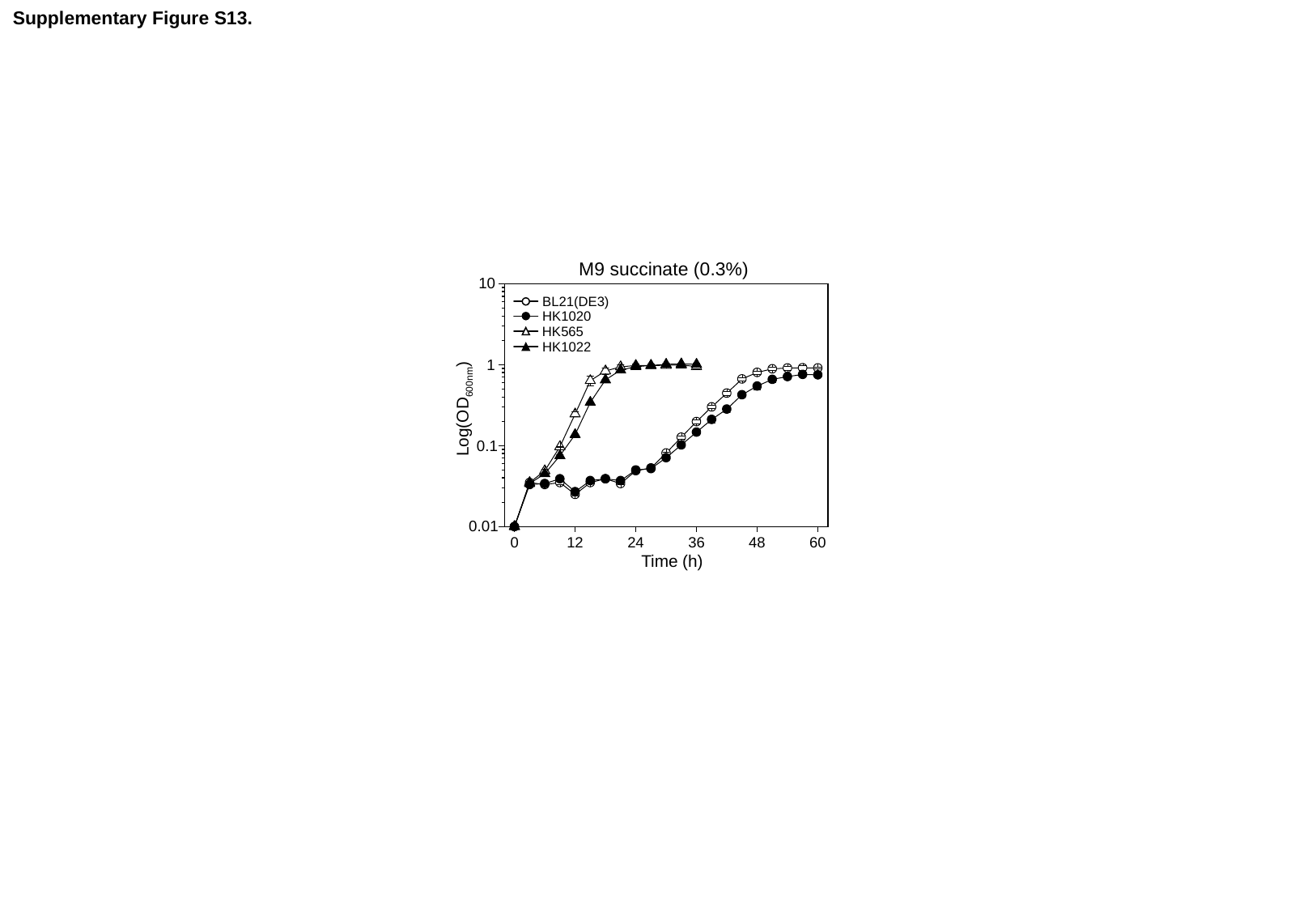

Supplementary Figure S13.
M9 succinate (0.3%)
10
1
0.1
0.01
BL21(DE3)
HK1020
HK565
HK1022
Log(OD600nm)
Time (h)

## Slide 14
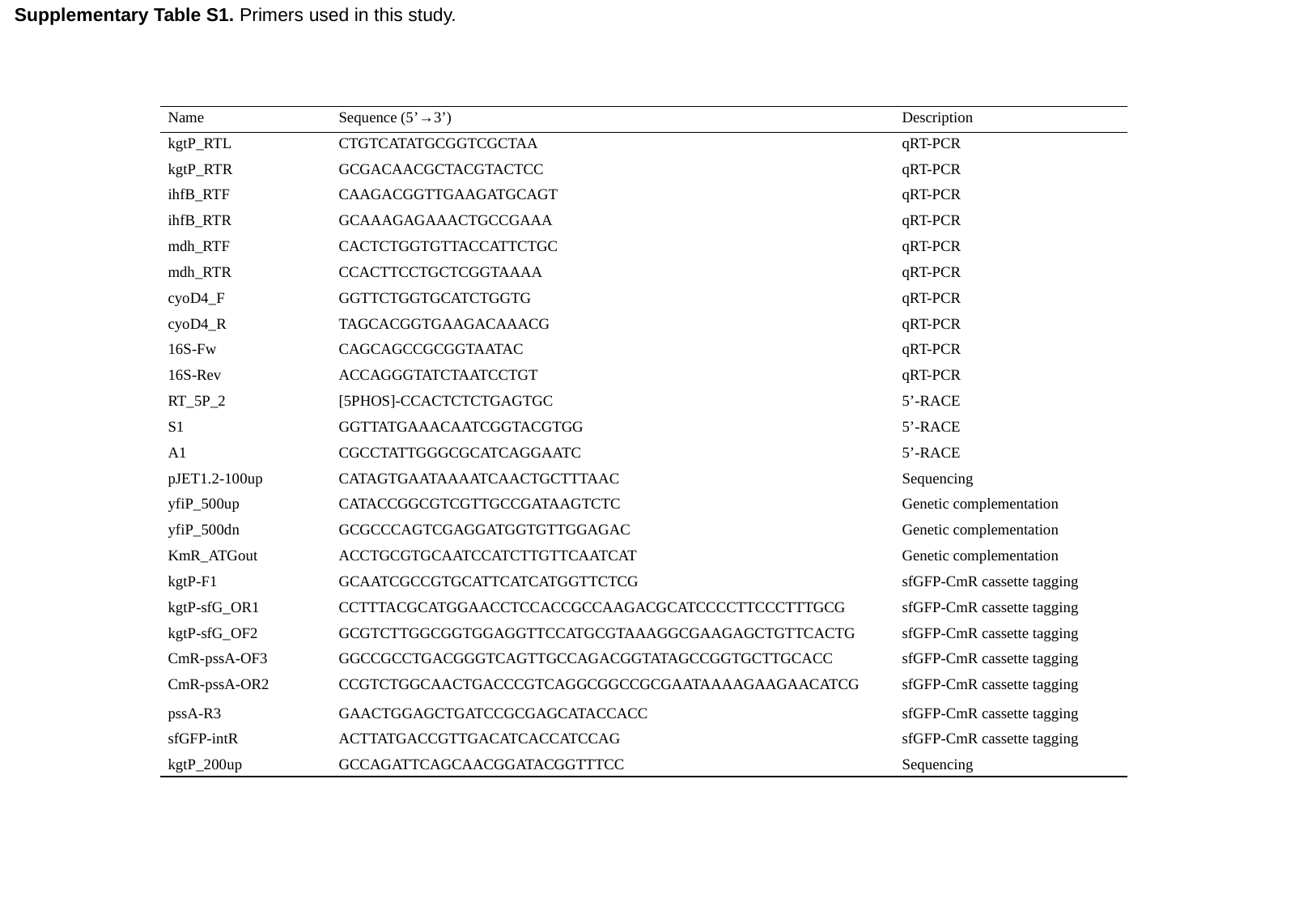

Supplementary Table S1. Primers used in this study.
| Name | Sequence (5’→3’) | Description |
| --- | --- | --- |
| kgtP\_RTL | CTGTCATATGCGGTCGCTAA | qRT-PCR |
| kgtP\_RTR | GCGACAACGCTACGTACTCC | qRT-PCR |
| ihfB\_RTF | CAAGACGGTTGAAGATGCAGT | qRT-PCR |
| ihfB\_RTR | GCAAAGAGAAACTGCCGAAA | qRT-PCR |
| mdh\_RTF | CACTCTGGTGTTACCATTCTGC | qRT-PCR |
| mdh\_RTR | CCACTTCCTGCTCGGTAAAA | qRT-PCR |
| cyoD4\_F | GGTTCTGGTGCATCTGGTG | qRT-PCR |
| cyoD4\_R | TAGCACGGTGAAGACAAACG | qRT-PCR |
| 16S-Fw | CAGCAGCCGCGGTAATAC | qRT-PCR |
| 16S-Rev | ACCAGGGTATCTAATCCTGT | qRT-PCR |
| RT\_5P\_2 | [5PHOS]-CCACTCTCTGAGTGC | 5’-RACE |
| S1 | GGTTATGAAACAATCGGTACGTGG | 5’-RACE |
| A1 | CGCCTATTGGGCGCATCAGGAATC | 5’-RACE |
| pJET1.2-100up | CATAGTGAATAAAATCAACTGCTTTAAC | Sequencing |
| yfiP\_500up | CATACCGGCGTCGTTGCCGATAAGTCTC | Genetic complementation |
| yfiP\_500dn | GCGCCCAGTCGAGGATGGTGTTGGAGAC | Genetic complementation |
| KmR\_ATGout | ACCTGCGTGCAATCCATCTTGTTCAATCAT | Genetic complementation |
| kgtP-F1 | GCAATCGCCGTGCATTCATCATGGTTCTCG | sfGFP-CmR cassette tagging |
| kgtP-sfG\_OR1 | CCTTTACGCATGGAACCTCCACCGCCAAGACGCATCCCCTTCCCTTTGCG | sfGFP-CmR cassette tagging |
| kgtP-sfG\_OF2 | GCGTCTTGGCGGTGGAGGTTCCATGCGTAAAGGCGAAGAGCTGTTCACTG | sfGFP-CmR cassette tagging |
| CmR-pssA-OF3 | GGCCGCCTGACGGGTCAGTTGCCAGACGGTATAGCCGGTGCTTGCACC | sfGFP-CmR cassette tagging |
| CmR-pssA-OR2 | CCGTCTGGCAACTGACCCGTCAGGCGGCCGCGAATAAAAGAAGAACATCG | sfGFP-CmR cassette tagging |
| pssA-R3 | GAACTGGAGCTGATCCGCGAGCATACCACC | sfGFP-CmR cassette tagging |
| sfGFP-intR | ACTTATGACCGTTGACATCACCATCCAG | sfGFP-CmR cassette tagging |
| kgtP\_200up | GCCAGATTCAGCAACGGATACGGTTTCC | Sequencing |
